# Synthesis and Investigation of Peptide–Drug Conjugates Comprising Camptothecin and a Human Protein‐Derived Cell‐Penetrating Peptide

**DOI:** 10.1111/cbdd.70051

**Published:** 2025-01-20

**Authors:** Isabella R. Palombi, Andrew M. White, Yasuko Koda, David J. Craik, Nicole Lawrence, Lara R. Malins

**Affiliations:** ^1^ Research School of Chemistry Australian National University Canberra Australian Capital Territory Australia; ^2^ Australian Research Council Centre of Excellence for Innovations in Peptide and Protein Science Australian National University Canberra Australian Capital Territory Australia; ^3^ Institute for Molecular Bioscience The University of Queensland Brisbane Queensland Australia; ^4^ Australian Research Council Centre of Excellence for Innovations in Peptide and Protein Science The University of Queensland Brisbane Queensland Australia

**Keywords:** camptothecin, cell‐penetrating peptide, cleavable linker, melanoma, peptide–drug conjugate

## Abstract

Drug targeting strategies, such as peptide–drug conjugates (PDCs), have arisen to combat the issue of off‐target toxicity that is commonly associated with chemotherapeutic small molecule drugs. Here we investigated the ability of PDCs comprising a human protein‐derived cell‐penetrating peptide—platelet factor 4‐derived internalization peptide (PDIP)—as a targeting strategy to improve the selectivity of camptothecin (CPT), a topoisomerase I inhibitor that suffers from off‐target toxicity. The intranuclear target of CPT allowed exploration of PDC design features required for optimal potency. A suite of PDCs with various structural characteristics, including alternative conjugation strategies (such as azide–alkyne cycloaddition and disulfide conjugation) and linker types (non‐cleavable or cleavable), were synthesized and investigated for their anticancer activity. Membrane permeability and cytotoxicity studies revealed that intact PDIP‐CPT PDCs can cross membranes, and that PDCs with disulfide‐ and protease‐cleavable linkers liberated free CPT and killed melanoma cells with nanomolar potency. However, selectivity of the PDIP carrier peptide for melanoma compared to noncancerous epidermal cells was not maintained for the PDCs. This study emphasizes the distinct role of the peptide, linker, and drug for optimal PDC activity and highlights the need to carefully match components when assembling PDCs as targeted therapies.

## Graphical abstract

Peptide–drug conjugates (PDCs) were produced by combining a cancer selective cell‐penetrating peptide (PDIP) with camptothecin (CPT). The PDCs crossed membranes and cleavable PDCs killed melanoma cells with nanomolar potency.

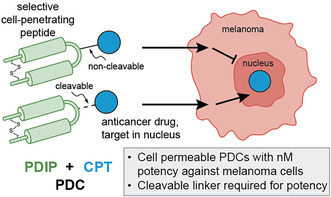

## 1 Introduction

Cancer poses a serious global health burden, with nearly 20 million new cases and 10 million deaths worldwide in 2022—statistics that are expected to increase in the coming decades (Ferlay et al. 2024; IARC 2024). Surgery, chemotherapy, radiotherapy, and immunotherapy are the conventional methods for treating the disease, but new therapies and treatment approaches will be required to address increasing cancer incidence (Rizvi et al. 2024). Many chemotherapeutic agents target rapidly dividing cells, resulting in systemic toxicity against healthy proliferative cells. This manifests as patient side effects, a hallmark of small molecule anticancer treatments (Hoppenz, Els‐Heindl, and Beck‐Sickinger 2020).

Camptothecin (CPT), originally isolated from the bark of the *Camptotheca accuminata* tree, is an alkaloid with antitumor activity against diverse cancer types (Botella and Rivero‐Buceta 2017; Wall et al. 1966). Its mechanism of action involves inhibition of topoisomerase I, an enzyme that relaxes supercoiled DNA during replication and transcription, leading to DNA damage and cell apoptosis (Chen and Liu 1994; Pommier 2006). Unfortunately, the clinical use of CPT has been hindered by off‐target toxicity, poor solubility of its rigid fused‐ring system and instability of the lactone moiety—which produces an inactive carboxylate species after hydrolysis at physiological pH (Botella and Rivero‐Buceta 2017). With these limitations in mind, a variety of CPT derivatives with structural modifications have been developed over the last few decades, ultimately resulting in three clinically approved analogues—irinotecan, topotecan, and belotecan (Venditto and Simanek 2010; Wang et al. 2023).

Recent research has examined alternative methods of improving CPT safety, for example by attaching the drug onto a cancer cell targeting device—such as an antibody (Conilh et al. 2023) or peptide (Fang and Wang 2022; Rizvi et al. 2024)—or by utilizing self‐assembling nanostructures (Botella and Rivero‐Buceta 2017) as drug carriers. The approval of trastuzumab deruxtecan (Enhertu®) in 2019, an antibody–drug conjugate (ADC) containing a CPT derivative payload attached to an anti‐HER2 monoclonal antibody (Ogitani et al. 2016; Xu et al. 2019), highlighted targeted drug delivery as a promising treatment approach for improving CPT safety. Although ADCs have the advantage of reducing the side effects of cytotoxic drugs, they are costly to produce, can have limited tumor penetration and can induce an immune response. Due to their smaller size, peptide–drug conjugates (PDCs) are often able to overcome the limitations of ADCs, while maintaining selectivity for specific intracellular targets (Wang et al. 2018, 2024). In a PDC approach, CPT has been paired with cell‐penetrating (El‐Sayed et al. 2019; Zhang et al. 2023) and cell‐targeting (Fuselier et al. 2003; Henne et al. 2006; Hou et al. 2022; Moody et al. 2004; Redko et al. 2017; Sun, Fuselier, and Coy 2004; Zhou et al. 2020, 2021, 2022;) peptides, with continued interest in the exploration of peptide scaffolds that utilize diverse drug delivery mechanisms.

**FIGURE 1:**
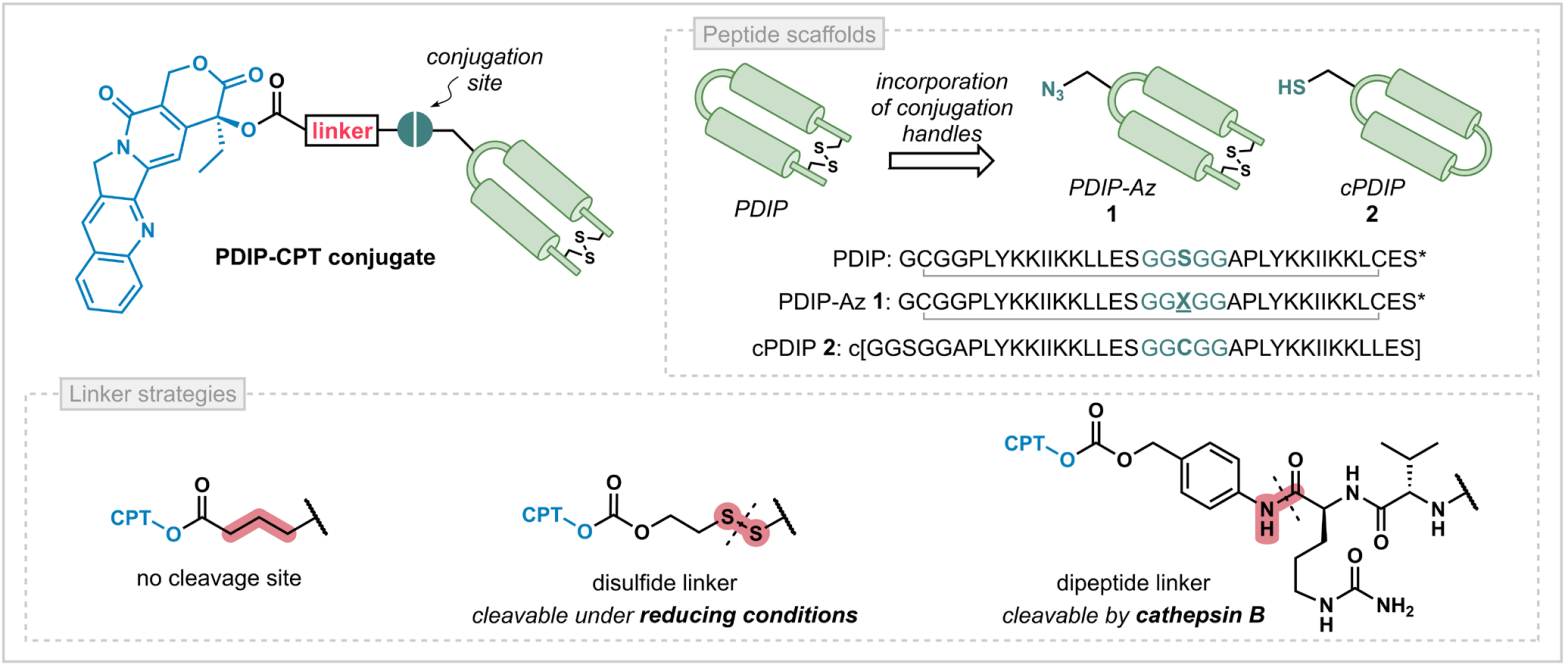
The proposed characteristics varied to generate a suite of PDIP‐CPT PDCs, including the peptide conjugation handle and linker type (colored pink). For cleavable linkers, the cleavage site is indicated with a dotted black line. For the peptide sequences, disulfide bonds are indicated with a grey line and the residues in the spacer region are colored teal; c[ ] = backbone cyclized; X = azidoalanine; *amidated C‐terminus.

We previously designed a human protein‐derived cell‐penetrating peptide (CPP) capable of delivering cargo into diseased cells via direct translocation across the plasma membrane (Lawrence et al. 2020; Palombi et al. 2023; Philippe et al. 2021). The peptide, platelet factor 4‐derived internalization peptide (PDIP; see Figure 1 for sequence), is a structural mimic of the amphipathic C‐terminal domain from the human defense protein platelet factor 4. PDIP comprises an alpha‐helical hairpin structure that is cyclized via a disulfide bond. Along with having CPP properties, it has low micromolar anticancer activity (Lawrence et al. 2020). In melanoma and leukemia cancer cells, this activity is attributed to disruption and impairment of the function of mitochondrial membranes. Furthermore, PDIP can selectively interact with and traverse negatively charged membranes, a property conferred by the cationic residues within its two helical domains (Lawrence et al. 2020). The exposure of anionic phosphatidylserine (PS) lipid headgroups on the surface of cancer cell membranes (Vallabhapurapu et al. 2015) provides a unique opportunity for the selective entry of a cationic CPP into cancer cells. Comparatively, healthy cells have neutrally charged outer membranes, due to PS lipids being maintained in the inner leaflet by flippase enzymes (Nagata et al. 2016; Seigneuret and Devaux 1984). Thus, a PDIP‐containing PDC could provide intracellular delivery of cytotoxic drugs into negatively charged cancer cells, while preventing interaction of the drug with healthy tissues.

PDIP has been shown to act as a tool to deliver peptide cargos into cancer cells (Lawrence et al. 2020; Philippe et al. 2021), but here we investigated its ability to selectively deliver a small molecule anticancer drug. CPT was identified as a model drug because its potent toxicity could be used to distinguish peptide versus drug cargo activity of PDCs against diseased cells, and its intranuclear target could be used to probe the requirement for release of CPT from the PDC. We hypothesized that PDIP‐CPT PDCs could improve the selectivity of the small molecule drug, and would likely require intracellular release of CPT from PDIP to allow CPT to inhibit topoisomerase I. Accordingly, we synthesized four PDIP‐CPT PDCs, incorporating distinct conjugation strategies and the ability to liberate the CPT drug cargo via reductive or enzymatic cleavage, with the aim of understanding how PDC components and design features influence biological activity, membrane permeability, and cellular uptake. This work demonstrates that PDIP‐CPT conjugates can cross membranes and kill melanoma cells with nanomolar potency when a traceless cleavable linker is included in the PDC design. However, selectivity of the PDIP carrier peptide for melanoma compared to noncancerous cells was not maintained for the CPT‐containing PDCs. The insights provided by this study highlight the distinct roles of the peptide, linker, and drug components and emphasize that PDC design is crucial for a successful targeted therapeutic approach.

## 2 Results and Discussion

### 2.1 Design of PDIP‐CPT Conjugates

A modular strategy for PDC synthesis was utilized to probe how specific components and design characteristics influence potency. Two PDIP analogues with different conjugation handles were proposed to understand the role of the peptide scaffold. The sequences and schematic representations of the peptide structures are shown in Figure 1 and they were synthesized as previously described (Lawrence et al. 2020; Palombi et al. 2023). The first analogue, PDIP‐Az (**1**), is similar to the parent peptide (PDIP) as it is cyclized via a disulfide bond. It contains an azide handle in the spacer region between the two helices, primed for drug conjugation via azide‐alkyne cycloaddition chemistry. The second analogue is a backbone cyclized variant containing only proteinogenic amino acids, herein referred to as cPDIP (**2**), providing a scaffold that does not require incorporation of bioorthogonal functionalities, such as an azide. The single cysteine residue in the spacer region enables macrocyclization via native chemical ligation during synthesis (Dawson et al. 1994), while also providing a reactive handle for downstream drug conjugation.

CPT‐linker constructs were designed to contain either an alkyne or activated disulfide moiety for PDC formation with the two PDIP analogues. Three alternative linkers, encompassing the region between CPT and the peptide, were proposed to identify whether CPT release is required to provide potent PDCs (Figure 1). A non‐cleavable linker is straightforward to install so is beneficial if drug and/or peptide activity is maintained following PDC construction. However, it is possible that this irreversible modification might prevent CPT from reaching or inhibiting topoisomerase I in the nucleus, requiring a linker that allows for traceless release of CPT from PDIP. Accordingly, two self‐immolative linkers were envisioned (Edupuganti, Tyndall, and Gamble 2021), bearing either a disulfide linkage that is reducible by intracellular glutathione (Henne et al. 2006; Kularatne et al. 2010) or a dipeptide moiety that is cleavable by the protease cathepsin B (Dubowchik et al. 2002) (Figure 1).

### 2.2 Modification of CPT With Cleavable or Non‐Cleavable Linkers and PDC Synthesis

**SCHEME 1:**
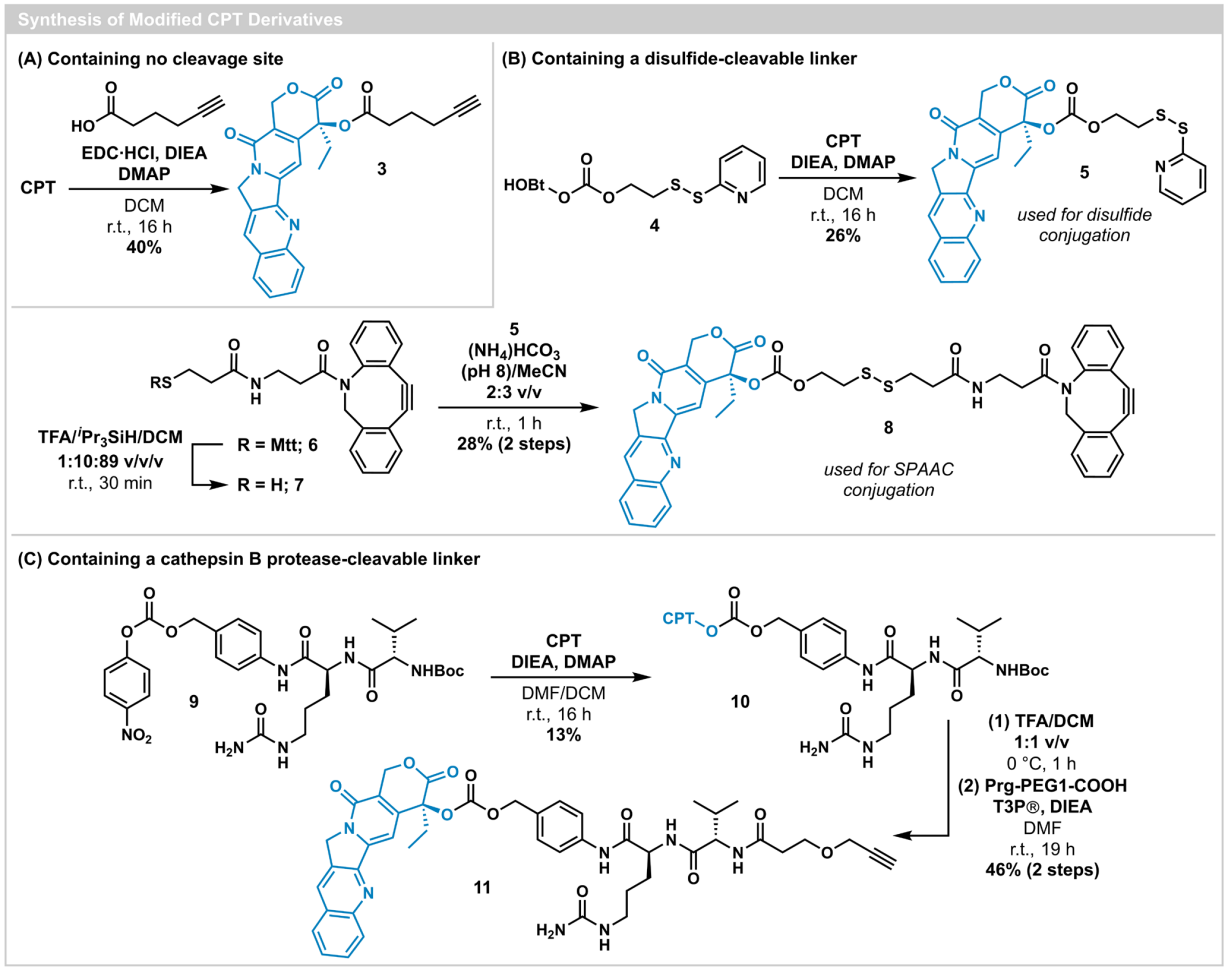
Modification of CPT to produce constructs with (A) a non‐cleavable linker, (B) a disulfide‐cleavable linker, and (C) a cathepsin B protease‐sensitive linker.

The construction of PDIP‐CPT PDCs first required modification of CPT to install the linkers and handles necessary for conjugation to peptides **1** and **2**. The hydroxyl group of CPT was chosen as an ideal site for linker attachment because modifications such as alkylation or acylation at this position are accessible and have been shown to improve stability of the lactone (Venditto and Simanek 2010; Zhao et al. 2000). CPT was thus modified at the tertiary alcohol to install a non‐cleavable linker by reacting it with 5‐hexynoic acid, in the presence of the coupling reagent EDC∙HCl, to produce **3** in 40% yield (Scheme 1A).

The synthesis of CPT with a cleavable disulfide linker began with treatment of HOBt‐activated compound **4** (Kularatne et al. 2010) with CPT under basic conditions and in the presence of DMAP, forming carbonate **5** in 26% yield (Scheme 1B). Compound **5** served the dual purpose of providing an activated disulfide for conjugation to thiol‐containing cPDIP (**2**) and a viable precursor to a second disulfide linker containing an alkyne handle for conjugation to PDIP‐Az (**1**). To accomplish the latter objective, activated disulfide **5** was reacted with thiol‐containing DBCO analogue **7**, following the acidic deprotection of methyltrityl‐protected precursor **6**. Disulfide exchange under mildly basic conditions (pH 8) afforded CPT‐linker construct **8** (28% yield over two steps) (Palombi et al. 2023).

The construction of a protease‐sensitive linker first involved installation of CPT onto commercially available Boc‐Val‐Cit‐PAB‐PNP (**9**) via a carbonate linkage (Scheme 1C). Removal of the Boc group on **10** allowed for subsequent amide formation at the deprotected amine with propargyl‐PEG1‐acid, aided by the coupling reagent T3P®, providing alkyne‐containing CPT‐linker construct **11** (46% yield over two steps).

**FIGURE 2:**
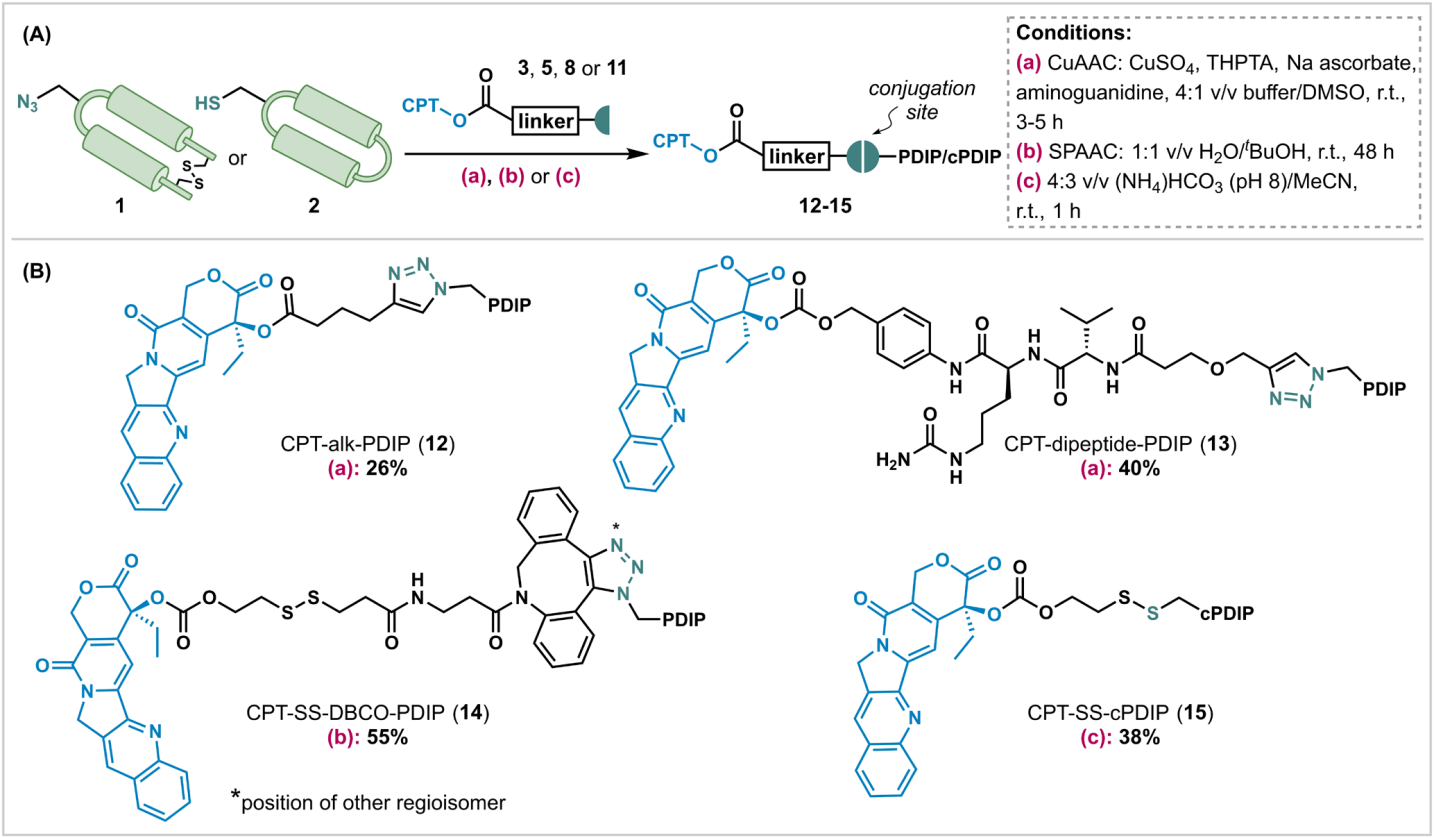
(A) General scheme and conditions for PDC synthesis. (B) Structures and yields of the four PDCs. The yields were determined using the molecular weight of the TFA salt, assuming all basic residues, the terminal amine (if present) and the quinoline moiety within CPT are protonated.

With CPT‐linker constructs in hand (**3**, **5**, **8**, and **11**), four water soluble PDCs were synthesized, the structures and yields of which are displayed in Figure 2 (see Supporting Information for analytical traces confirming product identity and purity). Two of these PDCs (**12**, **13**) were generated via a copper(I)‐catalyzed azide−alkyne cycloaddition (CuAAC) (Rostovtsev et al. 2002; Tornøe, Christensen, and Meldal 2002) reaction employing CuSO_4_, sodium ascorbate, and the water‐soluble ligand tris(3‐hydroxypropyltriazolylmethyl)amine (THPTA) (Hong et al. 2009). PDC **12** contains a non‐cleavable linker, formed by reacting terminal alkyne **3** with PDIP‐Az (**1**), and PDC **13** contains a protease‐sensitive linker, produced instead by reacting linker construct **11** with PDIP‐Az (**1**). Finally, two PDCs with a reducible disulfide linker were prepared, with each of the PDIP scaffold peptides. PDC **14** was generated via strain‐promoted azide−alkyne cycloaddition (SPAAC) (Agard, Prescher, and Bertozzi 2004) between cyclooctyne **8** and PDIP‐Az (**1**), to avoid the use of copper and excess reducing agents—which are required for CuAAC—in the presence of the disulfide‐cleavable functionality. In an alternative approach, PDC **15** was prepared by reacting reduced cPDIP (**2**) with the activated disulfide **5**, allowing for disulfide exchange under mildly basic conditions (pH 8).

### 2.3 Cytotoxicity and Hemolytic Activity of PDIP‐CPT Conjugates

PDIP is a membrane‐active CPP that has been shown to have low micromolar activity toward a melanoma cell line, with selectivity over noncancerous skin cells (Lawrence et al. 2020). Thus, the in vitro cytotoxicity of the four PDIP‐CPT conjugates was analyzed against melanoma (HT144) and noncancerous (HaCaT) cell lines. Cytotoxic activity of the PDCs was compared to the activity of CPT and the scaffold peptides—PDIP and c[A]PDIP—where the latter is a desulfurized variant of cPDIP (**2**) (see Supporting Information for synthetic details), produced to prevent dimerization of cPDIP via disulfide bonds formed between the sulfhydryl groups in physiological solutions.

#### 2.3.1 CPT Release From PDCs Is Required for Anticancer Activity

**FIGURE 3:**
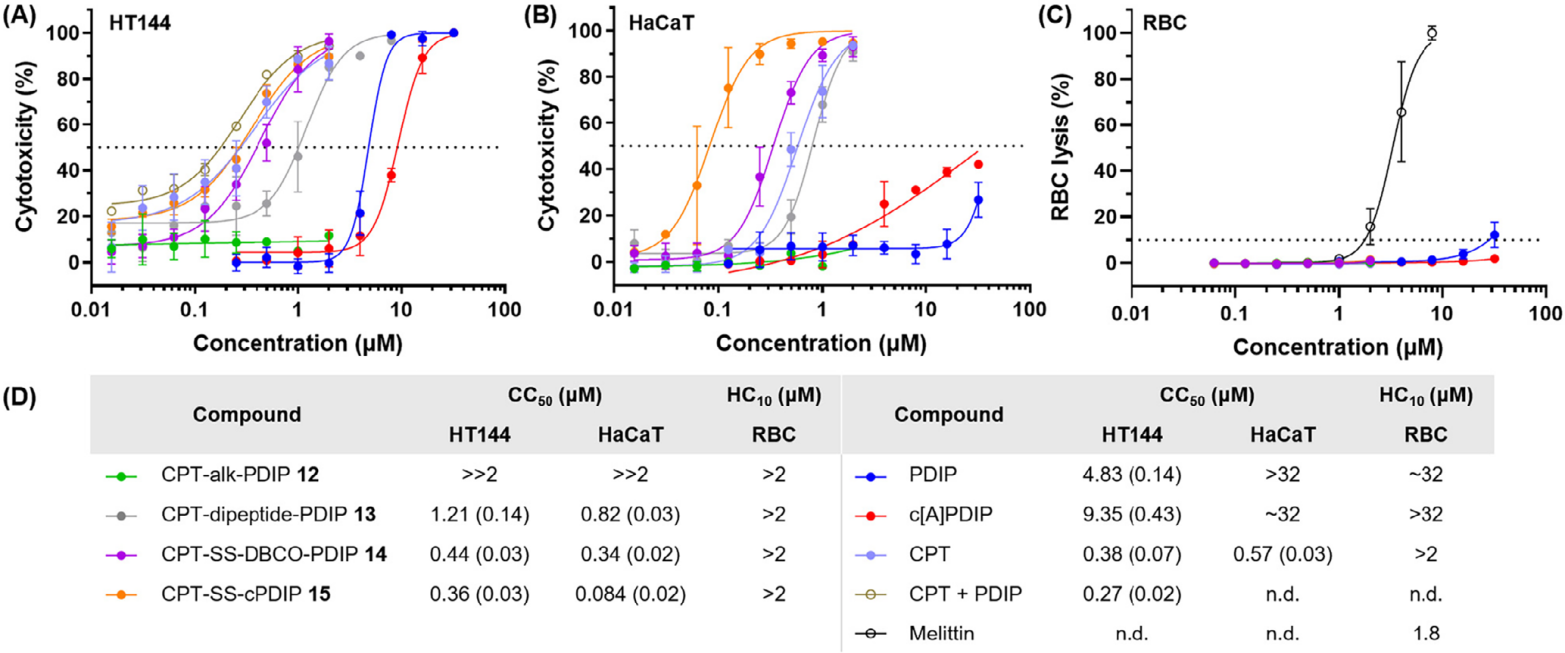
(A) and (B) Cytotoxicity of PDCs, CPT, and peptides against cultured HT144 (melanoma) and HaCaT (noncancerous) cells, following incubation for 72 h. Cell death was measured using resazurin, with 0.1% (v/v) Triton X‐100 as a control for 100% cell death. Data points are expressed as mean ± SD for at least two biological replicates. Dose response curves were fitted using [inhibitor] versus response with variable slope, Graphpad Prism v10. (C) Hemolysis of human RBCs (0.25% v/v) following incubation with PDCs, CPT, and peptides for 72 h. RBC lysis was determined by measuring released hemoglobin, with 0.1% (v/v) Triton X‐100 as a control for 100% cell lysis. Melittin was included as a membranolytic control. Data points are expressed as mean ± SD for two technical replicates. Dose response curves were fitted using [inhibitor] versus response with variable slope, Graphpad Prism v10. (D) Half maximal cytotoxicity concentration (CC_50_) and minimal hemolytic concentration (HC_10_) values of PDCs, CPT, and peptides. CC_50_ values are expressed as mean (SEM). The legend applies to all three graphs. c[A]PDIP is a desulfurized variant of cPDIP **2** (see Table S1) to prevent the presence of free thiol during assays. n.d., not determined.

Cytotoxicity testing of the four PDCs against the HT144 melanoma cell line, where cells were incubated with the treatments for 72 h, revealed nanomolar or low micromolar half maximal cytotoxicity concentration (CC_50_) values for the three cleavable PDCs **13** (1.21 μM), **14** (0.44 μM), and **15** (0.36 μM) (Figure 3A,D). In contrast, the non‐cleavable PDC **12** had no activity in the concentration range tested (16 nM—2 μM), supporting our hypothesis that release of CPT from PDIP is required before the drug can inhibit its intranuclear target, topoisomerase I. Finally, treatment of the melanoma cells with an equimolar mixture of unconjugated CPT and PDIP resulted in similar activity to CPT itself, indicating that while PDIP does not appear to enhance the activity of CPT, it probably does not interfere with the mechanism of action of the free drug or its ability to interact with topoisomerase I.

#### 2.3.2 PDC Potency Is Cargo Driven But Non‐Selective

The cleavable PDCs (**13**–**15**) had similar levels of cytotoxicity toward the melanoma cell line as CPT alone (CC_50_ = 0.38 μM), especially the conjugates with disulfide linkers (**14**–**15**) (Figure 3A,D). Comparatively, the two peptides were less potent (PDIP CC_50_ = 4.83 μM; c[A]PDIP CC_50_ = 9.35 μM), suggesting that CPT is the main component contributing to PDC activity at sublethal peptide concentrations. To further investigate whether the CPT cargo drives PDC potency, activity was examined following a shorter incubation time (24 h) but with the same concentration range (16 nM to 2 μM) where the PDCs but not PDIP were shown to be active after 72 h incubation (Figure 3A, Supporting Information, Figure S1). The absence of activity observed for CPT and the PDCs at concentrations below the effective range for PDIP and c[A]PDIP, and at a time point (24 h) that was too short to detect CPT toxicity (Supporting Information, Figure S1), confirms that the observed activity of PDCs at 72 h was driven by the CPT cargo. Comparison of PDIP and c[A]PDIP activity at concentrations up to 32 μM revealed similar activity ranges at both 24 h (Supporting Information, Figure S1) and 72 h (Figure 3A,D), which agrees with our previous observations of the rapid action of PDIP analogues against melanoma cells (Lawrence et al. 2020).

Although displaying favorable activity against the melanoma cell line, the cleavable PDCs (**13**–**15**) had similar toxicity against the control, noncancerous cell line (HaCaT) at nanomolar to low micromolar concentrations (Figure 3B,D). This was an unexpected result because PDIP and c[A]PDIP have CC_50_ ≥ 32 μM toward HaCaT cells. Therefore, it is possible that the addition of a hydrophobic drug and linker to the cationic peptide reduced the selectivity of PDIP for the anionic membranes present in cancer cells (see Supporting Information, Table S1, for relative hydrophobicity of PDIP and PDCs). However, since CPT itself is cell permeable, we cannot rule out the possibility that the observed toxicity resulted from gradual extracellular cleavage or degradation of PDCs **13**–**15** before cell internalization (Balamkundu and Liu 2023; Lei et al. 2021). Cleavable PDC **15** with the cPDIP scaffold was the most toxic PDC (CC_50_ = 84 nM against HaCaT, Figure 3B), suggesting that constraining the peptide via backbone cyclization may alter the interactions of charged and hydrophobic residues with membrane phospholipids required for selective cell entry.

To determine whether the PDCs showed toxicity toward cells with neutral membranes that were not immortalized for continuous culture, hemolysis assays were conducted with human RBCs (Figure 3C,D). The four PDCs (**12**–**15**) and CPT did not lyse RBCs within the same concentration range that they were active against the cell lines (16 nM—2 μM). PDIP displayed some hemolytic activity at the top concentration tested, with a minimal hemolytic concentration (HC_10_) of ~32 μM, whereas the control hemolytic peptide, melittin (Asthana, Yadav, and Ghosh 2004), had a HC_10_ of 1.8 μM and completely lysed the RBCs at 8 μM. Nevertheless, the lack of selectivity against a noncancerous cell line accentuates the importance of careful conjugate design to ensure that overall changes in the physiochemical properties (e.g., increased hydrophobicity) of the PDC compared to the peptide do not affect the safety window. Preserving the selectivity of PDIP for diseased cells over noncancerous cells after drug attachment is a high priority for future PDC design.

### 2.4 Membrane Permeability and Cell Uptake and Fate of PDIP‐CPT Conjugates

Membrane permeability, cellular uptake, and intracellular liberation of CPT were investigated alongside the cytotoxicity studies to provide an understanding of how PDC design influences compound internalization and potency.

#### 2.4.1 PDCs Are Membrane Permeable and Remain Intact

**FIGURE 4:**
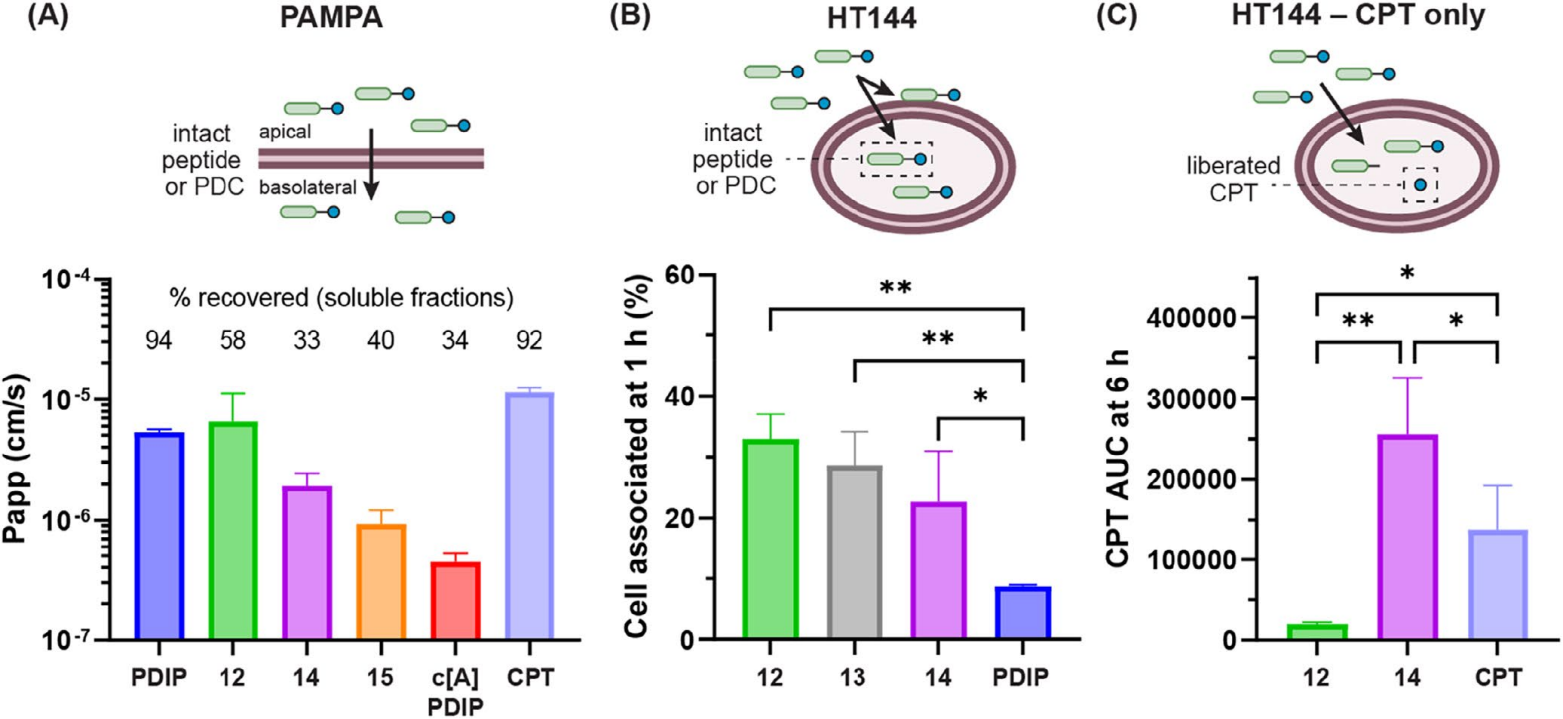
(A) Membrane permeability and cellular uptake of PDCs, scaffold peptides, and CPT. Compounds were added to PAMPA wells (4 μM) and incubated for 4 h. Supernatant from apical and basolateral sides of the membrane were collected and analyzed using LC–MS. The amount in the combined soluble fractions was compared to the starting amount to determine % recovery, and the apparent permeability (Papp) was calculated as before (Sevin et al. 2013). Data is expressed as mean ± SD from a single experiment with three technical replicates. (B) Internalization/cell membrane association of PDCs **12**–**14** and PDIP with HT144 cells. Cells were treated with PDIP or PDCs (4 μM) for 1 h, washed to remove extracellular compound, then extracted with 75% (v/v) MeCN in mQH_2_O (containing 1.75% (v/v) TFA), to recover soluble peptide or PDC from precipitated cell proteins. The relative amount of internalized peptide or PDC was determined by integrating area under the curve (AUC) for the [M + 6H]^6+^ *m/z* peak (for the intact mass) using time of flight–mass spectrometry (see Figure S2). The amount of internalized compound was compared to the AUC present when 4 μM of each analogue was added to untreated cells after addition of the extraction solution, to determine the percentage of internalized compound. Data is expressed as mean ± SD from a single experiment with three technical replicates for each treatment and control and was analyzed with ANOVA (Tukey multiple comparisons; **p < 0.01, *p < 0.05). (C) Detection of intracellular CPT following 6 h treatment of HT144 cells with CPT and PDCs **12** and **14**. Cells were treated with CPT, **12** or **14** (4 μM) for 6 h, washed to remove extracellular compound, and the relative amount of CPT was compared by determining AUC from targeted multiple reaction monitoring using tandem mass spectrometry (see Figure S3). Data is expressed as mean ± SD from a single experiment with three technical replicates for each treatment and was analyzed with ANOVA (Fisher multiple paired comparisons; **p < 0.01, *p < 0.05).

To determine whether intact peptides and PDCs could cross membranes, we used the parallel artificial membrane permeability assay (PAMPA) to report apparent permeability (Papp) because of passive movement through membranes, and mass spectrometric analysis of peptides and PDCs recovered from treated and washed HT144 cells to report on internalized compounds (passive or active transport). It should be noted that the latter method detects any sample that is associated with the harvested cells so can include both membrane‐associated and internalized compounds. Graphical representations of each assay are provided in Figure 4.

For the first assay, the PDCs (**12**–**15**), scaffold peptides and CPT (4 μM) were added to the apical side of PAMPA monolayers and incubated for 4 h. Analysis of the apical and basolateral sides of the membrane using liquid chromatography–mass spectrometry (LC–MS) revealed the Papp (movement from apical to basolateral fractions) and % recovery (amount of recovered sample in combined soluble fractions) for each compound (Figure 4A). PDIP, CPT, and PDCs **12** and **14** had high permeability (Papp ≥ 2 × 10^−6^ cm/s), highlighting that these PDCs maintain membrane permeability with the CPT cargo attached. However, both PDCs **12** and **14** had diminished recovery rates (58% and 33%, respectively) compared to CPT and PDIP (> 90%), suggesting that some membrane association or compound aggregation occurred during the assay. PDC **15** was less permeable (Papp ~9 × 10^−7^ cm/s), consistent with the lower permeability of the cyclic scaffold peptide c[A]PDIP (Papp ~4 × 10^−7^ cm/s). Furthermore, PDC **15** and c[A]PDIP had reduced recovery from the soluble fractions (40% and 34%, respectively), suggesting aggregation or membrane association during the assay. Reliable permeability data were not obtained for PDC **13** because of poor recovery of the PDC from soluble fractions, and degradation during the 4 h PAMPA experiment. Analysis of the degradation products revealed possible hydrolysis of **13** at the benzyl carbonate linkage, releasing CPT and CO_2_ from the dipeptide linker (see Supporting Information, Scheme S1). Negligible degradation was observed for the other two PDCs containing a carbonate linkage (**14** and **15**); these are probably more stable as the alkyl alcohol liberated upon carbonate hydrolysis is a poorer leaving group than the benzyl alcohol liberated from PDC **13**. Instability of the carbonate linkage within CPT conjugates containing a dipeptide linker has been previously reported (Walker et al. 2002), highlighting the potential incompatibility of this drug–linker combination for CPT PDCs. Considering the observed degradation of **13** in the cell‐free assay, it is also possible that extracellular degradation occurred during the cytotoxicity assays.

Representative cleavable (**13**–**14**) and non‐cleavable (**12**) PDCs, along with PDIP, were next examined to understand whether intact PDCs could be detected in HT144 cell extracts. Cells were treated with each compound (4 μM) for 1 h, followed by mass spectrometric analysis of washed and harvested cells. The area under the curve (AUC) values for each sample were compared to controls (untreated cells with compounds added post harvesting) to determine the total recovered PDC or peptide (see Supporting Information, Figure S2). A 1 h time point was chosen because it has been shown that PDIP enters cells within this time frame (Lawrence et al. 2020), and the concentration used to provide adequate detection (4 μM) is within the expected membrane‐active concentration range. The amount of recovered intact conjugate after 1 h for PDCs **12** (33%), **13** (29%), and **14** (23%) was significantly greater than that of the PDIP scaffold (9%) (Figure 4B), indicating rapid internalization and/or cell membrane association that may be aided by addition of the drug and linker to the peptide. This observed increase for the PDCs in the cell‐based assay was not consistent with the Papp determined from PAMPA. It is possible that active transport mechanisms contributed to PDC entry into the HT144 cells, but it is also likely that a proportion of the PDCs remained associated with cell membranes, which is consistent with the lower amounts recovered from the soluble fractions in the PAMPA experiment.

#### 2.4.2 Cleavable PDC 14 Releases CPT

To determine whether the CPT drug cargo is liberated from PDCs once inside melanoma cells, the amount of intracellular CPT was determined via tandem mass spectrometry. HT144 cells were treated with either CPT, the non‐cleavable PDC **12** or the disulfide‐cleavable PDC **14** for 6 h—a time point expected to allow for some linker cleavage on the basis of our previous work on disulfide linker fragmentation from PDIP conjugates (Palombi et al. 2023)—followed by the detection of relative amounts of CPT inside cells that had been washed and extracted as above (see Supporting Information, Figure S3). CPT was observed in the extract of HT144 cells treated with the disulfide‐cleavable PDC **14** (Figure 4C), suggesting that some intracellular cleavage had occurred within this time frame to release CPT from the PDIP carrier. In comparison, negligible amounts of CPT were detected for cells treated with the non‐cleavable PDC **12** (Figure 4C), indicating that the ester linkage to the drug is not hydrolyzed during this time frame (e.g., by intracellular esterases), in contrast to other reported potent non‐cleavable CPT conjugates (Hou et al. 2022). Since intact **12** was observed to cross membranes and enter cells (Figure 4A,B), the lack of activity for this PDC is likely not explained by the PDC being unable to enter the cell but rather by the inability for CPT to access its intranuclear target while tethered to a peptide.

Altogether, the permeability and uptake assays demonstrate that PDCs **12** and **14** maintained the high membrane permeability observed for PDIP and remained intact when inside and/or associated with melanoma cells. It is likely that the PDCs entered HT144 cells predominantly via a passive mechanism, but a portion of these conjugates may be aggregating/associating with the cell membrane. Association with membranes in a non‐selective manner (e.g., PAMPA) is consistent with the increased hydrophobicity of **12** and **14** relative to PDIP (see comparative HPLC retention times in Table S1), and their observed toxicity toward noncancerous HaCaT cells. Although PDC **13** degraded in the conditions of the PAMPA experiment, the intact mass was detected in treated HT144 cell extracts, suggesting that the conjugate did not completely degrade prior to cell entry/association in the shorter 1 h experiment. It is also possible that this PDC—containing a linker known for its hydrophobicity (Bargh et al. 2019; Jeffrey et al. 2006)—aggregates to a greater extent than the other cleavable PDCs (**14**–**15**) thereby affecting the ability of intact PDC or liberated CPT to enter cells.

## 3 Conclusions

In summary, a suite of CPT‐containing PDCs was successfully produced by conjugating CPT to two analogues of PDIP, a CPP derived from the innate defense molecule human platelet factor 4. The modular PDC design strategy enabled flexible installation of the CPT drug cargo using a collection of different conjugation strategies (CuAAC, SPAAC, and disulfide conjugation chemistry) and facilitated the synthesis of four PDCs in moderate yields. Three synthetically accessible linker technologies were explored including a non‐cleavable alkane, a disulfide‐cleavable linker, and a cathepsin B (dipeptide)‐cleavable linker. The intranuclear target of CPT enabled comparison of the three linker types and emphasized the crucial requirement for a cleavable linker when peptide attachment interferes with drug localization.

The combination of CPT, PDIP, and a cleavable linker produced PDCs with cargo driven and nanomolar activity against a melanoma cell line, with potency that was comparable to CPT alone. The three cleavable PDCs did not retain the selectivity for melanoma cells observed for the parent peptides, as they were toxic to the noncancerous HaCaT cell line. However, the PDCs were not hemolytic to human RBCs within the concentration range where they were active against HT144 and HaCaT cell lines, suggesting they do not exert an effect on neutral mammalian cell membranes at the tested concentrations.

Investigation of membrane permeability and cell uptake showed that PDIP is a highly membrane permeable peptide that can carry CPT cargo across membranes and was a superior carrier peptide relative to its backbone cyclic analogue, cPDIP. Despite the high apparent permeability of PDCs **12** and **14**, a degree of aggregation/membrane association likely affected the uptake of these PDCs into cells compared to the parent peptide. Intact PDCs were detected in extracts of HT144 cells after treatment for 1 h, even for PDC **13** with the dipeptide linker, which showed evidence of degradation over longer assays because of the unstable carbonate linkage. However, programmed drug release under reductive conditions was confirmed with liberated CPT observed for the disulfide‐cleavable PDC **14** after 6 h.

Future PDIP‐CPT PDCs should explore alternative linker types and conjugation strategies to improve PDC activity and selectivity. Cleavable linkers that are acid‐labile or iron(II)‐sensitive (Goldenberg et al. 2015; Spangler et al. 2018) and conjugation strategies such as oxime ligation (Lang and Chin 2014) are examples of alternative approaches that could be applied in the context of cancer treatment. The design of subsequent PDCs should take care to minimize hydrophobicity added to the peptide.

In conclusion, this study provided insight into the role of the peptide, linker, and drug components of PDCs for potential applications as targeted cancer therapies. PDIP‐CPT PDCs maintained the high membrane permeability of the carrier PDIP peptide, and liberation of the CPT drug cargo to access an intranuclear target was achieved through incorporating a disulfide‐cleavable linker. However, the loss of PDC selectivity for melanoma cells compared to the carrier PDIP peptide needs to be addressed when designing future PDCs, especially when the cargo is a cell‐permeable, cytotoxic drug.

## 4 Experimental Procedures

Detailed experimental and compound characterization data can be found in the Supporting Information file, along with figures, schemes, and tables.

## Ethics Statement

RBC lysis assays (using human RBC and serum) were performed in accordance with the University of Queensland Human Research Ethics Approval number 2022/HE000300.

## Conflicts of Interest

The authors declare no conflicts of interest.

## Supporting information


Data S1.

## Data Availability

The data that supports the findings of this study are available in the Supporting Information of this article.
