# Supplementary material for: Synthesis and Investigation of Peptide–Drug Conjugates Comprising Camptothecin and a Human Protein‐Derived Cell‐Penetrating Peptide
Source: Chem Biol Drug Des. 2025 Jan 20;105(1):e70051. doi: 10.1111/cbdd.70051 (PMC11747586; doi:10.1111/cbdd.70051)
Supplement: Supplementary file 1 — Data S1. [file CBDD-105-e70051-s001.pdf]

Supporting Information

Synthesis and Biological Evaluation of Peptide–Drug Conjugates Comprising a Human Protein-Derived Cell-Penetrating Peptide and the Anticancer Drug Camptothecin

Isabella R. Palombi,[a,b] Andrew M. White,[a,b] Yasuko Koda,[c,d] David J. Craik,[c,d] Nicole Lawrence,*[c,d] Lara R. Malins*[a,b]

[a] Research School of Chemistry, Australian National University, Canberra, ACT 2601, Australia

[b] Australian Research Council Centre of Excellence for Innovations in Peptide and Protein Science, Australian National University, Canberra, ACT 2601, Australia

[c] Institute for Molecular Bioscience, The University of Queensland, Brisbane, QLD 4072, Australia

[d] Australian Research Council Centre of Excellence for Innovations in Peptide and Protein Science, The University of Queensland, Brisbane, QLD 4072, Australia

*Email: lara.malins@anu.edu.au; n.lawrence@imb.uq.edu.au.

Contents

Supplementary Figures 3
General Chemistry Procedures........ 6
CPT-alkyne (**3**) synthesis 6
Disulfide linkers (**5** and **8**) synthesis........ 7
Dipeptide linker (**11**) synthesis 9
Synthesis and purification of cPDIP (**2**) and c[A]PDIP........ 10
General procedure: CuAAC conjugation........ 12
CPT-SS-DBCO-PDIP (**14**) synthesis 14
CPT-SS-cPDIP (**15**) synthesis........ 15
Cell culture........ 15
Cell toxicity........ 16
Red blood cell (RBC) lysis 16
Parallel artificial membrane permeability assay (PAMPA) 17
Compound internalization/association with HT144 cells 17
CPT detection inside HT144 cells 18
^{1}H and ^{13}C{^{1}H} NMR........ 19
References........ 30

Supplementary Figures

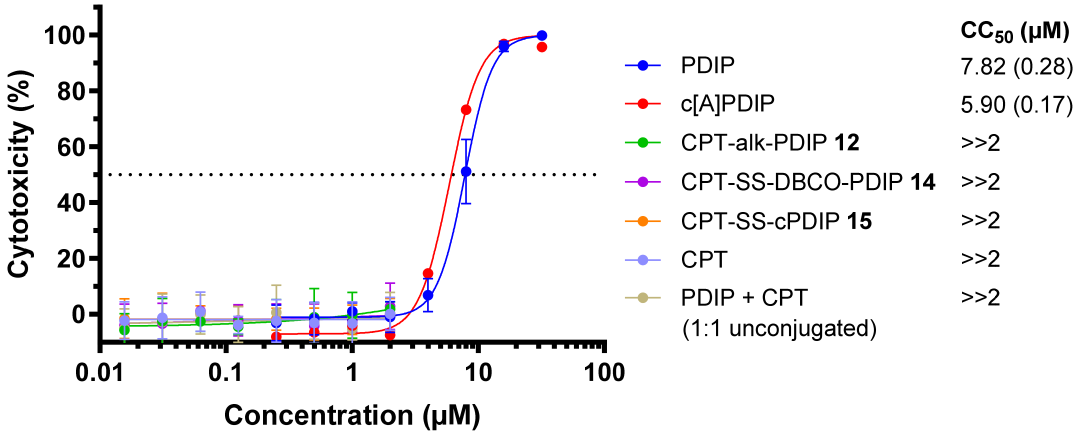

Figure S1. Cytotoxicity of PDCs, CPT, and peptides against HT144 cultured melanoma cells, following incubation for 24 h. Cell death was measured using resazurin, with 0.1% (v/v) Triton X-100 as a control for 100% cell death. Data points are expressed as mean ± SD for at least two biological replicates. CC_{50} values are expressed as mean (SEM). Note: CPT-dipeptide-PDIP was not included in this study. c[A]PDIP is a desulfurized variant of cPDIP **2** (see Table S1) to prevent the presence of free thiol during assays.

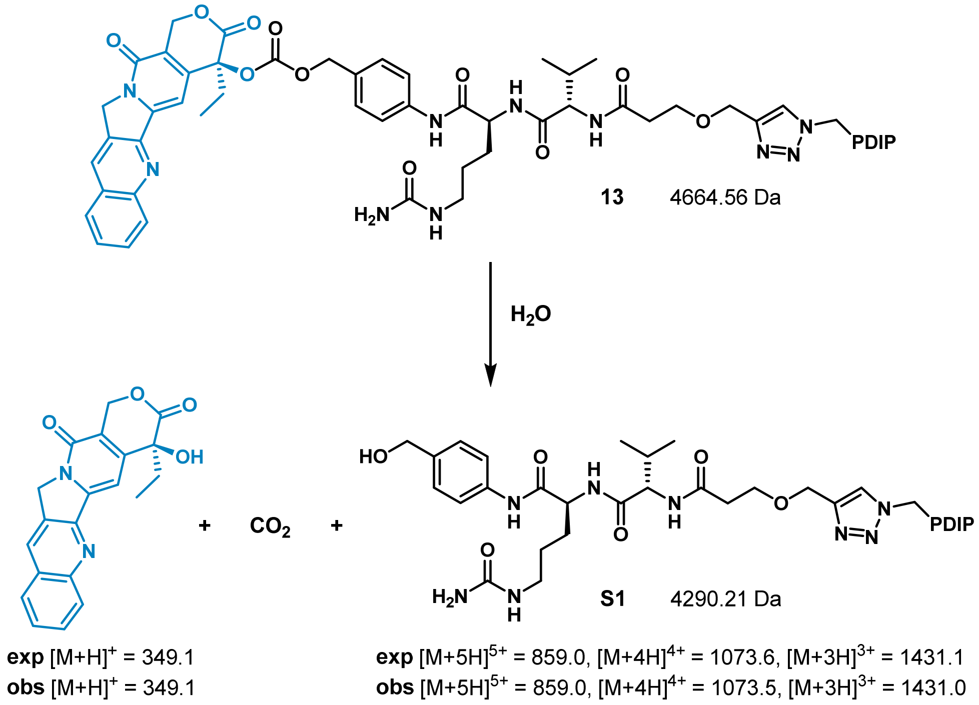

Scheme S1. Proposed degradation of PDC **13** observed in the cell-free environment for PAMPA. Hydrolysis of the carbonate functional group and subsequent decarboxylation releases CPT, CO_2, and alcohol containing **S1**. The *m/z* values obtained from LC–MS analysis in support of this breakdown pathway are shown underneath each product.

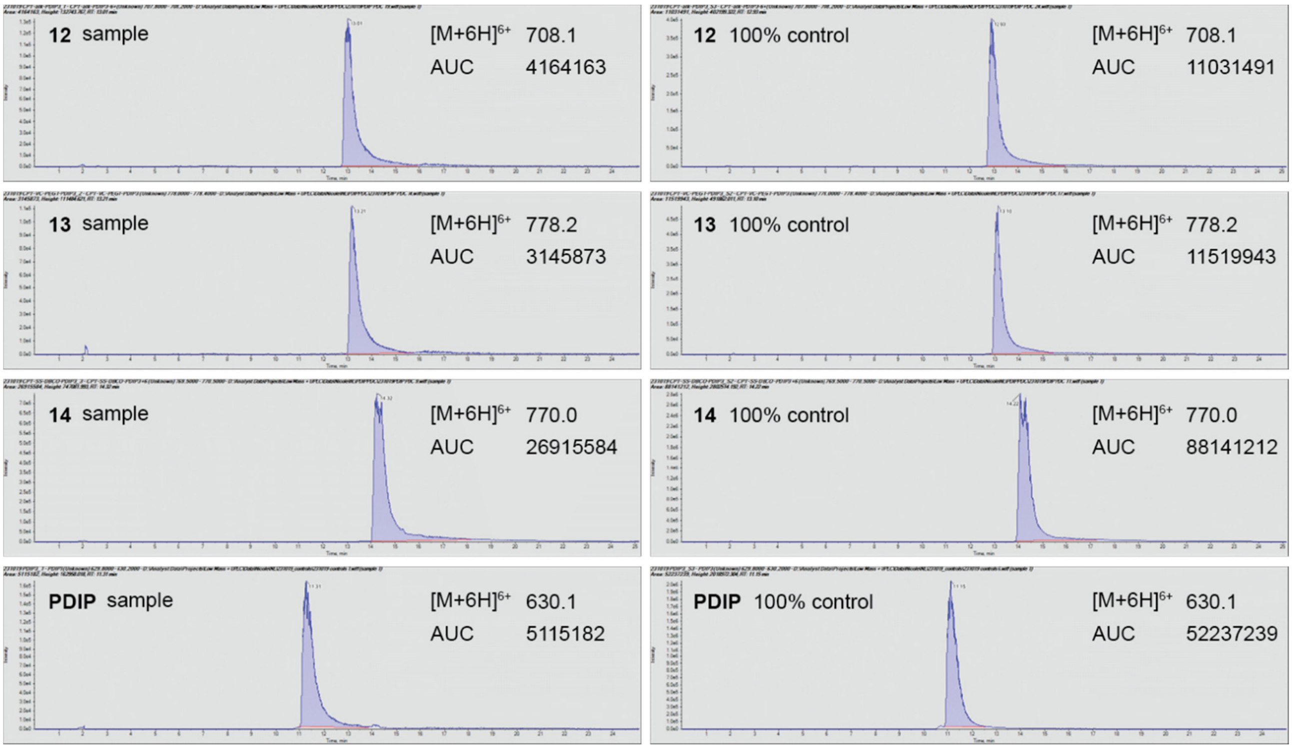

Figure S2. Representative MS extracted ion chromatograms for PDCs **12-14** and PDIP following extraction of treated and control HT144 cells. Samples and controls were analyzed using a Qstar elite TOF-MS with a 2% gradient containing 0.1% (v/v) formic acid in H_2O against 0.1% (v/v) formic acid in 90% (v/v) $MeCN/H_2O$. The area under the curve (AUC) for the $[M+6H]^{6+}$ *m/z* peak for each compound was determined using Sciex MultiQuant software.

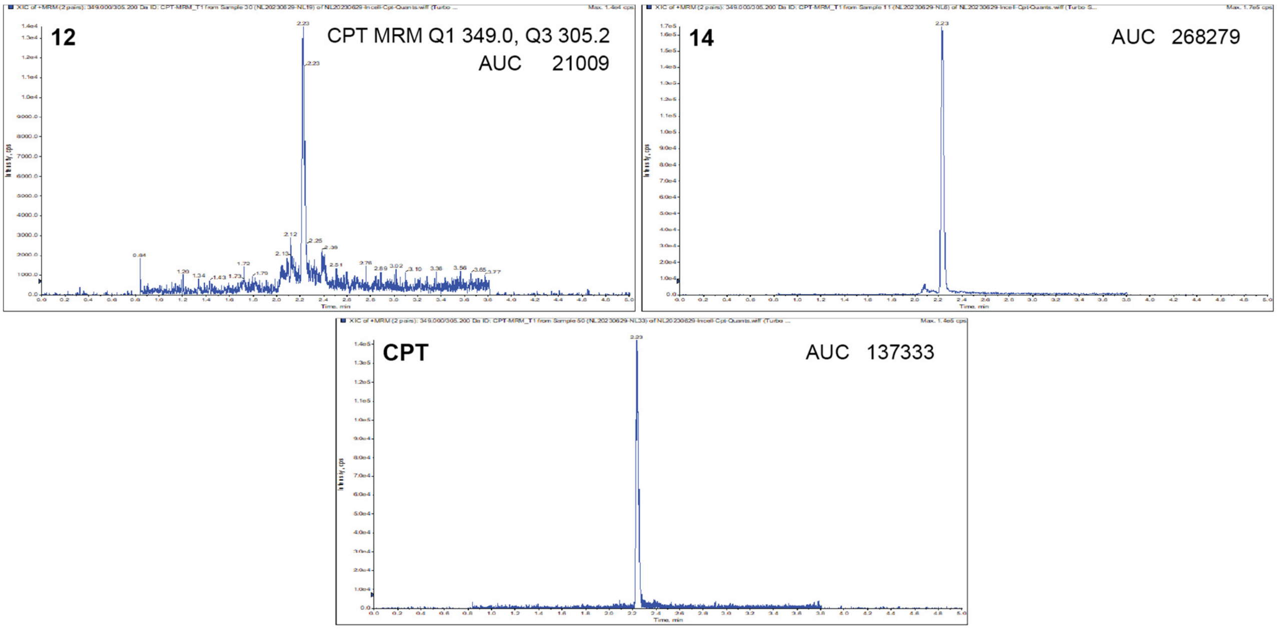

Figure S3. Representative extracted ion chromatograms for CPT using targeted multiple reaction monitoring (MRM), following treatment of HT144 cells with PDCs **12** and **14**, or CPT for 6 h. Samples were analyzed using a Sciex QTRAP® 6500+ MS coupled to an Exion UPLC system. Scans were performed with low resolution in quadrupole 1 (Q1) and unit resolution in quadrupole 3 (Q3), with the following MRM parameters: Q1 (*m/z*) 349.0, Q3 305.2, collision energy 40, and declustering potential 80. The area under the curve (AUC) for CPT was determined for each sample using Sciex MultiQuant software.

Table S1. Sequences/structures and properties of PDIP peptide analogues and PDCs.

Peptide or PDC	Sequence or structure	MW (Da)[a]	MW (Da) TFA salt[b]	Charge at pH 7.4[c]	RT (min)[d]
PDIP	GCGGPLYKKIIKKLLESGGSGGAPLYKKIIKKLCES*	3775.6	4801.8	9	14.8
PDIP-Az **1**	GCGGPLYKKIIKKLLESGGXGGAPLYKKIIKKLCES*	3800.6	4826.8	9	16.8
cPDIP **2**	c[GGCGGAPLYKKIIKKLLESGGSGGAPLYKKIIKKLLES]	3898.8	4810.9	8	18.1
c[A]PDIP[e]	c[GG**A**GGAPLYKKIIKKLLESGGSGGAPLYKKIIKKLLES]	3866.7	4778.9	8	18.0
CPT-alk-PDIP **12**	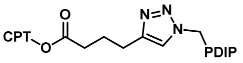	4243.1	5383.3	9	18.8
CPT-dipeptide-PDIP **13**	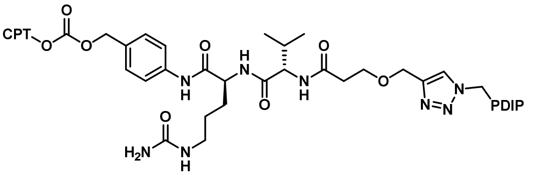	4664.6	5804.8	9	19.1
CPT-SS-DBCO-PDIP **14**	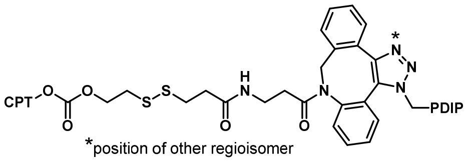	4615.6	5755.8	9	20.9, 21.2 (two regio-isomers)
CPT-SS-cPDIP **15**	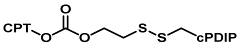	4349.2	5375.4	8	21.1

X = azidoalanine

* denotes an amidated C-terminus

c[ ] = backbone cyclized

[a] Molecular weight was calculated from the contributions of amino acids, linker, and drug components, using ChemDraw v23. For peptides and PDCs containing a cyclic disulfide, the oxidized molecular weight is provided.

[b] Calculated TFA salt molecular weight includes the mass of TFA counterions resulting from protonation of basic residues and the N-terminal amine (if present) within the peptides (for peptides and PDCs), and protonation of the quinoline moiety within CPT (for PDCs), after HPLC purification with acidic eluent (0.1% TFA). The molecular weight assumes no salt-bridge formation between amino acids.

[c] The calculated charge of each compound at pH 7.4 (physiological pH similar to assay conditions), assuming no salt-bridge formation between amino acids.

[d] Retention time (RT) was determined using analytical reverse-phase HPLC with a gradient of 5% to 65% MeCN (0.1% TFA) over 30 min. More hydrophobic compounds have a longer RT.

[e] A desulfurized variant of cPDIP was employed in cytotoxicity assays to prevent the presence of a free thiol during assays.

General Chemistry Procedures

^{1}H and $^{13}C\{^{1}H\}$ NMR spectra were recorded on a Bruker AVANCE spectrometer (400/800 MHz) at 298 K, in the solvents specified. $CDCl_3$ was treated with K_2CO_3(s) and 4 Å molecular sieves prior to use. For ^{1}H NMR and ^{13}C NMR spectra, signals arising from the residual protio-form and deutero-form of the solvent, respectively, were used as an internal reference. These correspond to δ_H 7.26 and δ_C 77.16 for $CDCl_3$, and δ_H 3.31 and δ_C 49.00 for CD_3OD. ^{1}H NMR data are recorded as follows: chemical shift (δ) [multiplicity, coupling constant(s) *J* (Hz), relative integral] where multiplicity is defined as: s = singlet; d = doublet; t = triplet; m = multiplet or combinations of the above. Coupling constants are quoted to the nearest 0.1 Hz.

High-resolution mass spectrometry (HRMS) was carried out using positive ESI on a Waters Synapt G2-Si mass spectrometer. Preparative high-performance liquid chromatography (HPLC) was performed on a Waters 600 Controller with a Waters 717 plus Autosampler and a Waters 2996 Photodiode Array Detector running Empower Pro Empower 3 software. Liquid chromatography–mass spectrometry (LC–MS) was performed a Shimadzu LCMS-2050 mass spectrometer with a Shimadzu LC40Dx3 UHPLC system. Analytical HPLC was performed on an Agilent 1100 Analytical HPLC with an Agilent Zorbax SB-C18 column or a Shimadzu LCMS-2020 instrument with a Phenomenex 5 µm C18 / 300 Å / 150 × 2 mm LC column. Linear gradients of water (solvent A) and MeCN (solvent B) were used for LC–MS, preparative and analytical HPLC with solvents containing 0.1% TFA for HPLC or 0.01% formic acid for LC–MS. Any deviations from the above are specified in the corresponding protocol. Dry solvents were obtained from a Glass Contour solvent purification system.

Peptide and PDC masses for yields were determined on a Mettler Toledo UMX2 microbalance to 0.001 mg accuracy. PDIP (formerly known as cPF4PD), cPDIP and c[A]PDIP were synthesized using published protocols;[1–3] PDIP-Az was manufactured by Wuxi AppTec or Mimotopes. Fmoc-azido-alanine and Boc-Val-Cit-PAB-PNP were manufactured by Combi-Blocks, other amino acids were purchased from GL Biochem or AK Scientific, PEG1-alkyne from BroadPharm and VA-044 from Fujifilm Wako Chemicals.

CPT-alkyne (3) synthesis

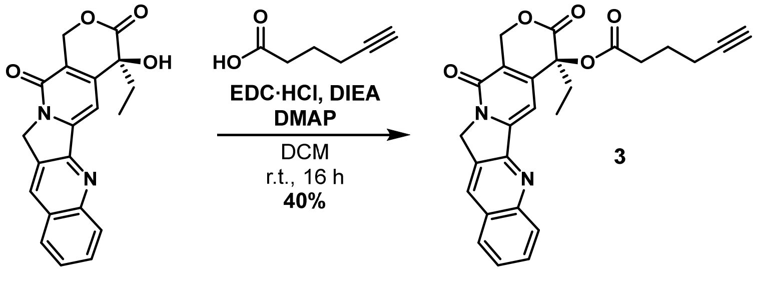

Following a procedure adapted from Li *et al.*:[4] A magnetically stirred mixture of camptothecin (CPT) (50 mg, 0.14 mmol, 1.0 equiv.), 5-hexynoic acid (40 µL, 0.36 mmol, 2.5 equiv.) and DIEA (125 µL, 0.724 mmol, 3.0 equiv.) in DCM (2 mL) maintained under an atmosphere of nitrogen was treated with EDC·HCl (69 mg, 0.36 mmol, 2.5 equiv.) and DMAP (8.5 mg, 70 µmol, 0.5 equiv.). The mixture was stirred at room temperature for 16 h. The mixture was diluted with chloroform (30 mL) and the organic phase was washed sequentially with saturated aqueous $NaHCO_3$ (10 mL) and brine (10 mL). The organic phase was dried (Na_2SO_4), filtered and concentrated under reduced pressure to give a crude product that was subjected to purification by reverse-phase semi-preparative HPLC (40 to 90% MeCN over 20 min) to afford **3** (31 mg, 40%) as a bright-yellow solid (TFA salt).

^{1}H NMR: (400 MHz, $CDCl_3$) δ 8.44 (s, 1H), 8.26 (d, *J* = 8.2 Hz, 1H), 7.96 (d, *J* = 8.2 Hz, 1H), 7.90 – 7.82 (m, 1H), 7.74 – 7.65 (m, 1H), 7.29 (s, 1H), 5.69 (d, *J* = 17.2 Hz, 1H), 5.41 (d, *J* = 17.2 Hz, 1H), 5.35 – 5.29 (m, 2H), 2.76 – 2.56 (m, 2H), 2.36 – 2.23 (m, 3H), 2.21 – 2.09 (m, 1H), 2.02 (t, *J* = 2.6 Hz, 1H), 1.93 – 1.83 (m, 2H), 0.99 (t, *J* = 7.5 Hz, 3H) ppm; **^{13}C NMR:** (101 MHz, $CDCl_3$) δ 172.3, 167.6, 157.5, 152.4, 148.9, 146.3, 146.3, 131.5, 131.0, 129.6, 128.6, 128.4, 128.4, 128.3, 120.4, 96.5, 83.2, 76.0, 69.6, 67.2, 50.1, 32.6, 32.0, 23.3, 17.8, 7.7 ppm; **HRMS:** (ESI+) *m/z* observed 465.1428, calculated for $C_{26}H_{22}N_2O_5Na$ $[M+Na]^+$ 465.1421.

Disulfide linkers (5 and 8) synthesis

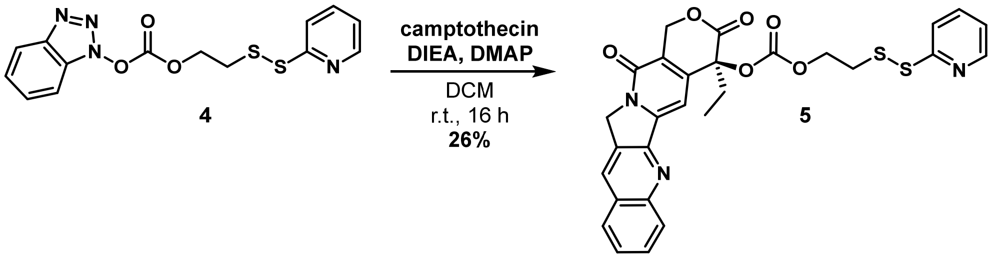

A magnetically stirred mixture of CPT (25 mg, 72 µmol, 1.0 equiv.) and carbonate **4**[5] (37 mg, 0.11 mmol, 1.5 equiv.) in DCM (5 mL) maintained under an atmosphere of nitrogen was treated with DIEA (35 µL, 0.20 mmol, 2.8 equiv.) and DMAP (2.2 mg, 18 µmol, 0.25 equiv.). The mixture was stirred at room temperature for 16 h, then concentrated under a stream of nitrogen and purified by reverse-phase semi-preparative HPLC (40 to 70% MeCN over 20 min) to afford **5** (15 mg, 26%) as a yellow solid (2 x TFA salt).

^{1}H NMR: (400 MHz, $CDCl_3$) δ 8.61 – 8.56 (m, 1H), 8.47 (s, 1H), 8.27 (d, *J* = 8.6 Hz, 1H), 8.00 – 7.91 (m, 3H), 7.90 – 7.84 (m, 1H), 7.75 – 7.68 (m, 1H), 7.42 (s, 1H), 7.33 – 7.27 (m, 1H), 5.70 (d, *J* = 17.3 Hz, 1H), 5.40 (d, *J* = 17.3 Hz, 1H), 5.37 – 5.32 (m, 2H), 4.40 – 4.27 (m, 2H), 3.11 (t, *J* = 6.3 Hz, 2H), 2.33 – 2.10 (m, 2H), 1.02 (t, *J* = 7.5 Hz, 3H) ppm; **^{13}C NMR:** (101 MHz, $CDCl_3$) δ 167.3, 158.9, 157.6, 153.5, 151.8, 148.4, 147.0, 146.5, 146.1, 140.7, 132.2, 131.5, 129.1, 128.7, 128.6, 128.5

(2C), 122.2, 122.1, 120.5, 97.5, 78.2, 67.0, 66.2, 50.5, 37.6, 32.0, 7.7 ppm; **HRMS:** (ESI+) *m/z* observed 562.1104, calculated for $C_{28}H_{24}N_3O_6S_2$ $[M+H]^+$ 562.1101.

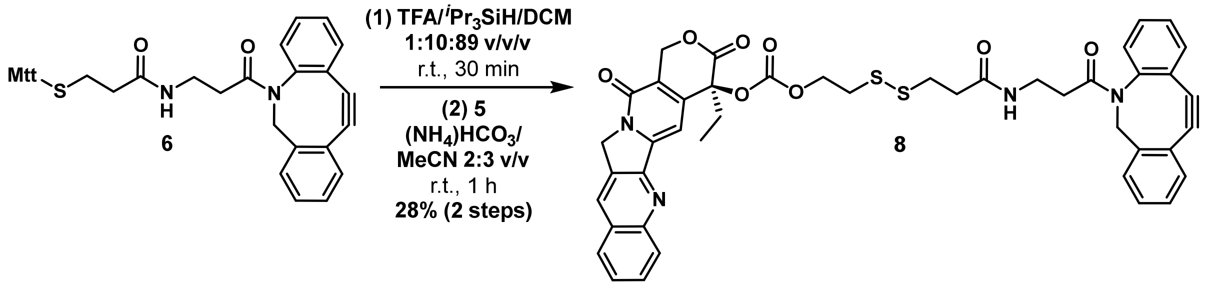

Compound **6**[6] (3.7 mg, 6.0 µmol, 1.5 equiv.) was suspended in a mixture of TFA/^{i}Pr$_3$SiH/DCM (1:10:89 v/v/v, 0.5 mL total) and magnetically stirred at room temperature for 30 min. The mixture was concentrated under a stream of nitrogen and the resulting residue was redissolved in argon purged MeCN (1.5 mL). This solution was transferred to a flask containing **5** (TFA salt, 3.0 mg, 3.8 µmol, 1.0 equiv.), followed by the addition of an argon purged aqueous ammonium bicarbonate solution (200 mM, pH 8, 1 mL). The mixture was magnetically stirred at room temperature for 1 h, then concentrated under a stream of nitrogen. Purification with reverse-phase semi-preparative HPLC (40 to 95% MeCN over 20 min) afforded **8** (1.0 mg, 28% over two steps) as a yellow solid (TFA salt).

^{1}H NMR: (400 MHz, $CDCl_3$) δ 8.45 – 8.41 (m, 1H), 8.27 – 8.22 (m, 1H), 7.98 – 7.93 (m, 1H), 7.87 – 7.82 (m, 1H), 7.72 – 7.61 (m, 2H), 7.44 – 7.27 (m, 8H), 6.47 – 6.39 (m, 1H), 5.70 (apparent dd, *J* = 17.3, 2.4 Hz, 1H), 5.39 (d, *J* = 17.3 Hz, 1H), 5.34 – 5.30 (m, 2H), 5.10 (d, *J* = 13.9 Hz, 1H), 4.44 – 4.29 (m, 2H), 3.66 (apparent dd, *J* = 13.9, 4.0 Hz, 1H), 3.39 – 3.26 (m, 2H), 2.91 (t, *J* = 6.7 Hz, 2H), 2.82 – 2.76 (m, 2H), 2.52 – 2.38 (m, 3H), 2.34 – 2.09 (m, 2H), 2.02 – 1.91 (m, 1H), 1.01 (t, *J* = 7.4 Hz, 3H) ppm; **HRMS:** (ESI+) *m/z* observed 815.2216, calculated for $C_{44}H_{39}N_4O_8S_2$ $[M+H]^+$ 815.2204.

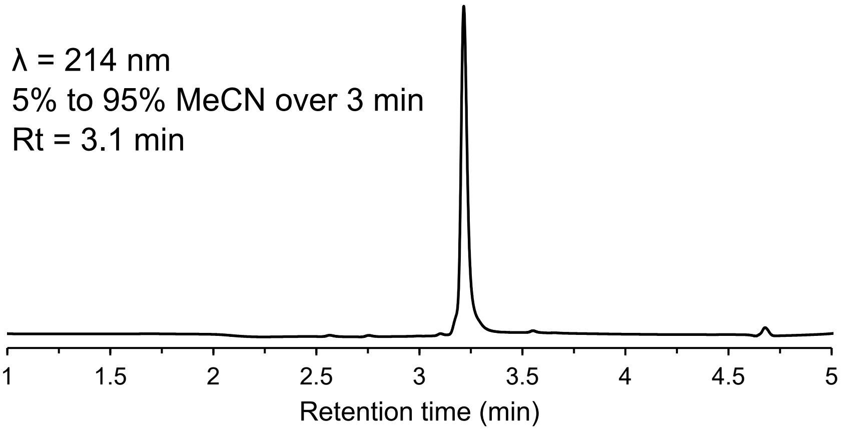

Dipeptide linker (11) synthesis

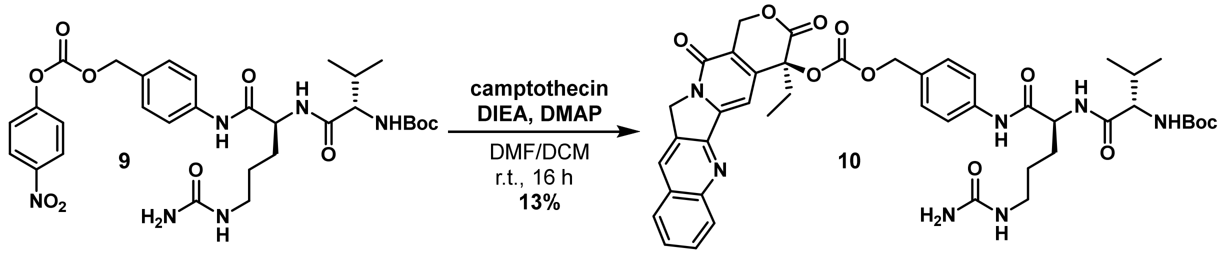

A magnetically stirred mixture of Boc-Val-Cit-PAB-PNP (**9**, 40 mg, 62 μmol, 1.0 equiv.) and CPT (30 mg, 86 μmol, 1.4 equiv.) in DMF (0.75 mL) and DCM (0.75 mL) maintained under an atmosphere of nitrogen was treated with DIEA (22 μL, 0.25 mmol, 4.0 equiv.) and DMAP (1.9 mg, 16 μmol, 0.25 equiv.). The mixture was stirred at room temperature for 16 h, then concentrated under a stream of nitrogen and purified by reverse-phase semi-preparative HPLC (40 to 90% MeCN over 20 min) to afford **10** (7.9 mg, 13%) as a light-yellow solid (TFA salt).

^{1}H NMR: (800 MHz, $CDCl_3$) *four N–Hs not observed* δ 8.98 (br s, 1H), 8.41 (s, 1H), 8.23 (d, *J* = 8.5 Hz, 1H), 7.94 (d, *J* = 8.5 Hz, 1H), 7.85 (apparent t, *J* = 7.4 Hz, 1H), 7.68 (apparent t, *J* = 7.4 Hz, 1H), 7.50 – 7.41 (m, 2H), 7.41 – 7.34 (m, 2H), 7.25 – 7.19 (m, 2H), 5.65 (d, *J* = 16.7 Hz, 1H), 5.36 (d, *J* = 16.7 Hz, 1H), 5.28 (s, 2H), 5.10 (d, *J* = 12.4 Hz, 1H), 4.95 (d, *J* = 12.4 Hz, 1H), 4.64 – 4.53 (m, 1H), 4.00 – 3.90 (m, 1H), 3.25 – 3.08 (m, 2H), 2.28 – 2.21 (m, 1H), 2.17 – 2.06 (m, 2H), 1.95 – 1.83 (m, 1H), 1.76 – 1.64 (m, 1H), 1.60 – 1.50 (m, 2H), 1.40 (s, 9H), 1.00 – 0.90 (m, 9H) ppm; **^{13}C NMR:** (201 MHz, $CDCl_3$) *one carbon signal not observed (or overlapping with another signal)* δ 173.3, 170.3, 167.9, 161.3, 157.5, 156.7, 153.7, 152.0, 148.7, 146.5, 146.2, 138.0, 131.8, 131.2, 130.8, 129.5, 129.3 (2C), 128.6, 128.4, 128.4, 120.4 (2C), 120.2, 96.8, 81.0, 78.1, 70.3, 67.2, 60.8, 53.6, 50.3, 39.8 (assigned from ^{1}H–^{13}C HSQC), 32.0, 30.6, 29.3, 28.4 (3C), 25.7, 19.4, 18.1, 7.7 ppm; **HRMS:** (ESI+) *m/z* observed 854.3729, calculated for $C_{44}H_{52}N_7O_{11}$ $[M+H]^+$ 854.3719.

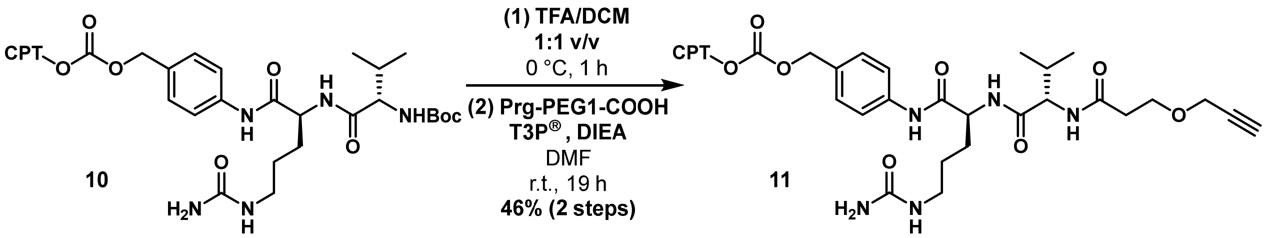

Compound **10** (TFA salt, 4.9 mg, 5.1 μmol, 1.0 equiv.) was suspended in a mixture of TFA/DCM (1:1 v/v, 0.2 mL total) and magnetically stirred at 0 °C (ice bath) for 1 h, after which the mixture was concentrated under a stream of nitrogen followed by vacuum. The resulting residue was redissolved in DMF (0.5 mL) and treated with propargyl-PEG1-acid (1.3 mg, 10 μmol, 2.0 equiv.), T3P® (50% in EtOAc, 9.7 μL, 15 μmol, 3.0 equiv.) and DIEA (6.0 μL, 31 μmol, 6.0 equiv.). The mixture was magnetically stirred at room temperature for 16 h, after which additional propargyl-PEG1-acid (1.3 mg), T3P® (9.7 μL) and DIEA (6.0 μL) were added to the reaction. The mixture was stirred at

room temperature for a further 3 h, then concentrated under a stream of nitrogen and subjected to purification by reverse-phase semi-preparative HPLC (35 to 75% MeCN over 20 min) to afford **11** (2.3 mg, 46% over two steps) as a light-yellow solid (TFA salt).

^{1}H NMR: (800 MHz, CD_3OD) *six N–Hs not observed* δ 8.59 (s, 1H), 8.15 (d, *J* = 8.3 Hz, 1H), 8.08 – 8.02 (m, 1H), 7.91 – 7.85 (m, 1H), 7.72 (apparent t, *J* = 7.1 Hz, 1H), 7.48 (d, *J* = 8.2 Hz, 2H), 7.35 (s, 1H), 7.27 (d, *J* = 8.2 Hz, 2H), 5.62 (d, *J* = 16.4 Hz, 1H), 5.47 (d, *J* = 16.4 Hz, 1H), 5.32 – 5.22 (m, 2H), 5.14 – 5.05 (m, 2H), 4.44 – 4.40 (m, 1H), 4.22 – 4.18 (m, 1H), 4.13 (t, *J* = 2.4 Hz, 2H), 3.81 – 3.75 (m, 2H), 3.23 – 3.16 (m, 1H), 3.12 – 3.07 (m, 1H), 2.82 (t, *J* = 2.4 Hz, 1H), 2.59 – 2.53 (m, 2H), 2.28 – 2.22 (m, 1H), 2.21 – 2.15 (m, 1H), 2.13 – 2.08 (m, 1H), 1.86 – 1.80 (m, 1H), 1.71 – 1.64 (m, 1H), 1.60 – 1.48 (m, 2H), 1.02 (t, *J* = 7.4 Hz, 3H), 0.97 (t, *J* = 7.2 Hz, 6H) ppm; **^{13}C NMR:** (201 MHz, CD_3OD) δ 174.2, 173.8, 172.1, 169.4, 162.4, 158.9, 154.9, 153.3, 149.6, 148.0, 147.8, 140.0, 133.3, 132.0, 131.9, 130.8, 130.2 (2C), 130.0, 129.9, 129.7, 129.2, 121.0 (2C), 120.9, 97.6, 80.4, 79.5, 76.1, 71.3, 67.8, 67.0, 60.6, 58.9, 54.8, 51.6, 40.4 (assigned from ^{1}H–^{13}C HSQC), 37.2, 32.2, 31.7, 30.4, 27.8, 19.8, 18.7, 8.0 ppm; **HRMS:** (ESI+) *m/z* observed 864.3568, calculated for $C_{45}H_{50}N_7O_{11}$ $[M+H]^+$ 864.3563.

Synthesis and purification of cPDIP (2) and c[A]PDIP

Assembly and backbone cyclization of cPDIP

Following a previously reported procedure from Lawrence *et al.*:[3] cPDIP was synthesized as a linear peptide hydrazide on Fmoc-NHNH-chlorotrityl resin[7,8] using standard automated Fmoc solid-phase chemistry (Symphony, Protein Technologies Inc). The peptide hydrazide was deprotected and cleaved from the resin using a mixture of TFA/H_2O/$^{i}Pr_3SiH$ (95:2.5:2.5 v/v/v) and collected by precipitating with ice-cold ether. The crude peptide was subsequently purified by reverse-phase preparative HPLC using a Shimadzu system and Phenomenex Gemini C18 column with a gradient of solvent B (90% MeCN/water, 0.05% TFA v/v) against solvent A (water, 0.05% TFA v/v).

The lyophilized linear peptide hydrazide—bearing an N-terminal cysteine residue—was backbone cyclized *via* intramolecular native chemical ligation.[9] To convert the C-terminal hydrazide to a thioester, the peptide (1.0 equiv.) was prepared as a 3 mM solution in Gn·HCl (6 M, pH <3), with 4-mercaptophenylacetic acid (200 mM) and acetyl acetone (9 mM, 3.0 equiv.),[10] and stirred at room temperature for 4 h. Head-to-tail cyclization was achieved by diluting the reaction to produce a 0.5 mM peptide solution in Gn·HCl (6 M), NaH_2PO_4 (100 mM) and TCEP (50 mM). The mixture was adjusted to pH 7 and stirred overnight at room temperature. The cyclized peptide was purified by reverse-phase preparative HPLC as above.

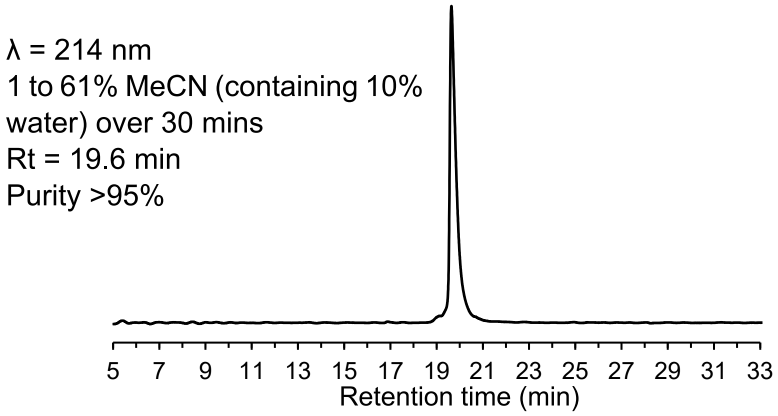

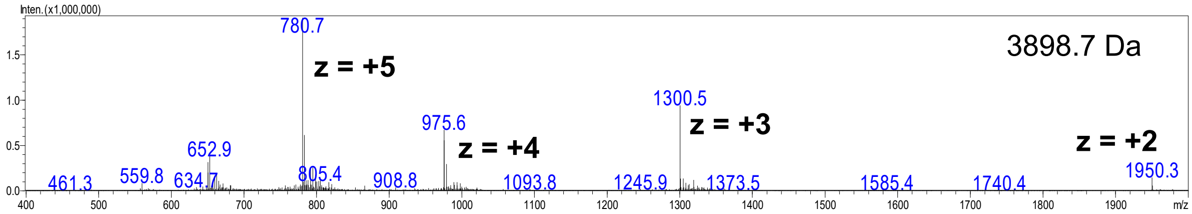

Desulfurization of cPDIP

cPDIP (**2**) was treated with TCEP (0.25 M), Gn·HCl (3 M), NaH_2PO_4 (50 mM), reduced glutathione (10 mM) and VA-044 (50 mM). The mixture was adjusted to pH 6.5, purged with argon, and incubated overnight at 65 °C.[11] The cyclized peptide was purified by reverse-phase preparative HPLC as above.

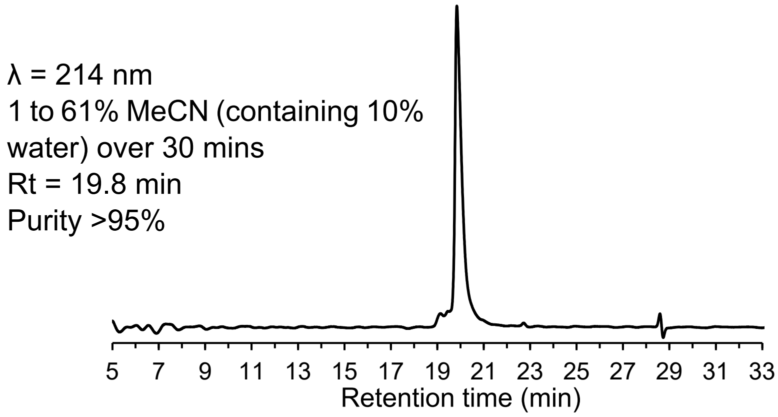

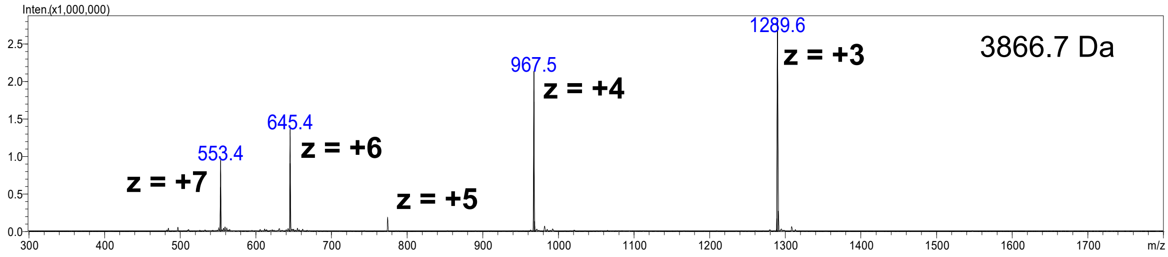

General procedure: CuAAC conjugation

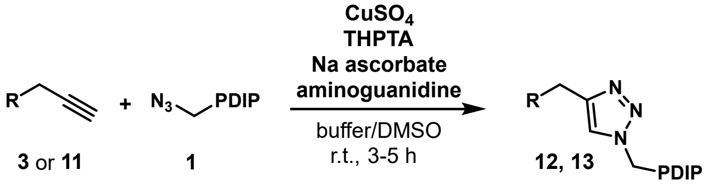

Following a procedure adapted from Hong *et al.*:[12] All reagent stock solutions were prepared in water, unless otherwise specified, to the following concentrations: aminoguanidine hydrochloride 100 mM, copper(II) sulfate 20 mM, tris(3-hydroxypropyltriazolylmethyl)amine (THPTA) 50 mM, sodium ascorbate 100 mM (freshly prepared), ethylenediaminetetraacetic acid (EDTA) 100 mM and drug-alkyne (**3** or **11**) 20 mM (in DMSO).

PDIP-Az (**1**, 1.0 equiv.) was dissolved in 100 mM sodium phosphate buffer (prepared by mixing 100 mM Na_2HPO_4 with 100 mM NaH_2PO_4 until pH = 7) to produce a final peptide concentration of 200 µM (based on total reaction volume including reagents to be added). An aliquot of DMSO was included to make the conjugation reaction mixture 20% organic/80% aqueous by volume. The following reagents were then added sequentially to the reaction vial and magnetically stirred after each new addition: aminoguanidine hydrochloride (5 mM[i], 25 equiv.), drug-alkyne (400 µM, 2.0 equiv.), copper(II) sulfate (100 µM, 0.5 equiv.) pre-mixed with THPTA (500 µM, 2.5 equiv.), and sodium ascorbate (5 mM, 25 equiv.). The conjugation mixture was magnetically stirred at room temperature for 3 – 5 h. The reaction was quenched with EDTA (5.0 equiv.) and purified by reverse-phase semi-preparative HPLC. Reported yields were determined by weight on a microbalance and were calculated using the mass of the TFA salt of the product (assuming all basic residues, the N-terminal amine and the quinoline moiety within CPT are protonated).

*CPT-alk-PDIP (**12**)*

Prepared with PDIP-Az **1** and CPT-alkyne **3** on a 0.44 µmol scale (2.1 mg peptide). Purification with semi-preparative HPLC (20 to 50% MeCN over 40 min) yielded **12** (0.60 mg, 26%) as a light-yellow solid.

[i] Concentration of each component refers to the final concentration in the reaction mixture

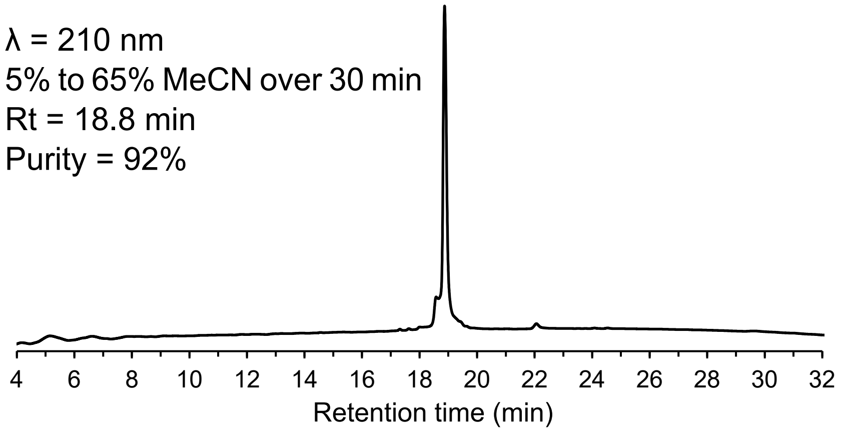

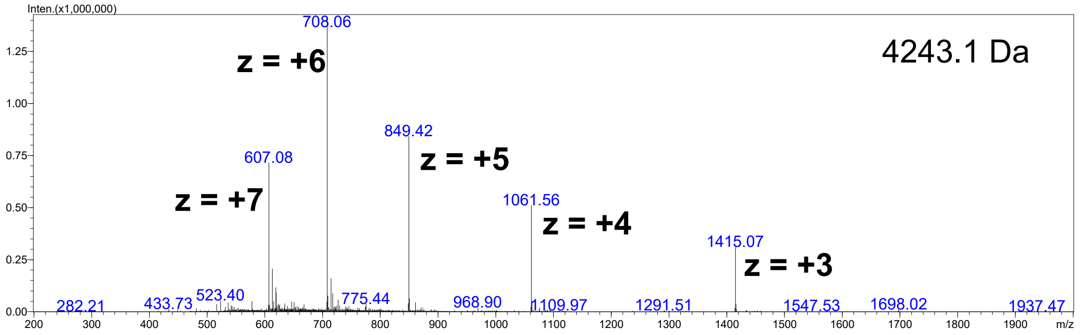

*CPT-dipeptide-PDIP (**13**)*

Prepared with PDIP-Az **1** and CPT-dipeptide-PEG1-alkyne **11** on a 0.31 μmol scale (1.5 mg peptide). Purification with semi-preparative HPLC (30 to 50% MeCN over 40 min) yielded **13** (0.73 mg, 40%) as a light-yellow solid.

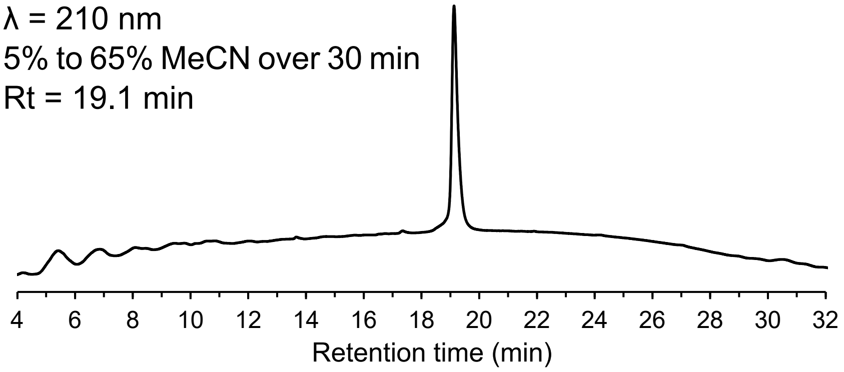

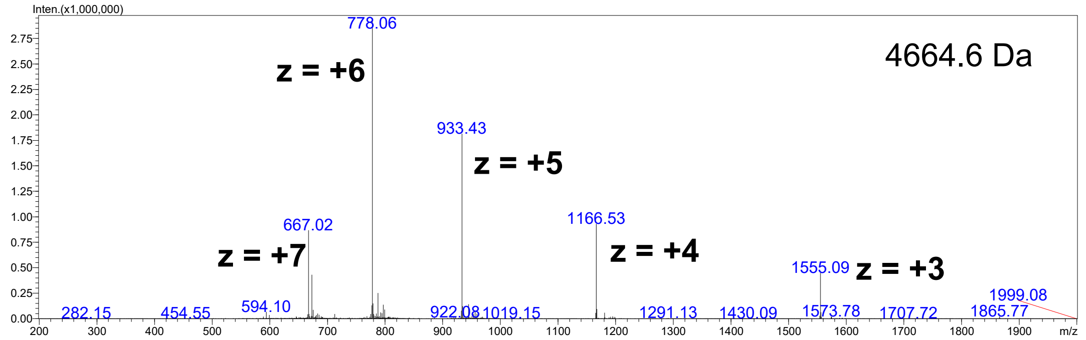

CPT-SS-DBCO-PDIP (14) synthesis

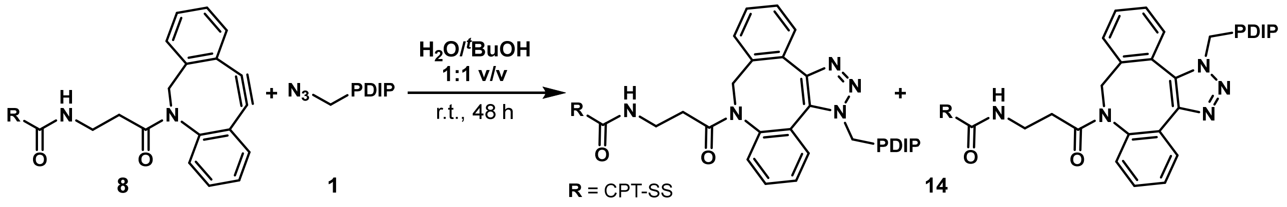

Following a procedure adapted from Lau *et al.*:[13] PDIP-Az **1** (3.8 mg, 0.79 µmol, 1.0 equiv.) and CPT-SS-DBCO **8** (1.0 mg, 1.2 µmol, 1.6 equiv.) were dissolved in water/*t*-butanol (1:1 v/v, 1 mL total). The mixture was stirred for 48 h to produce two regioisomers that were purified by reverse-phase semi-preparative HPLC (30 to 60% MeCN over 40 min) to afford **14** (2.48 mg, 55% for the combined regioisomers) as a light-yellow solid. Reported yield was determined by weight on a microbalance and was calculated using the mass of the TFA salt of the product (assuming all basic residues, the N-terminal amine and the quinoline moiety within CPT are protonated).

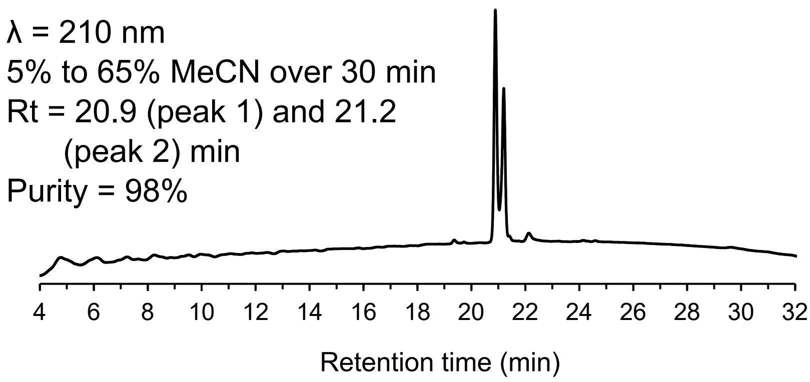

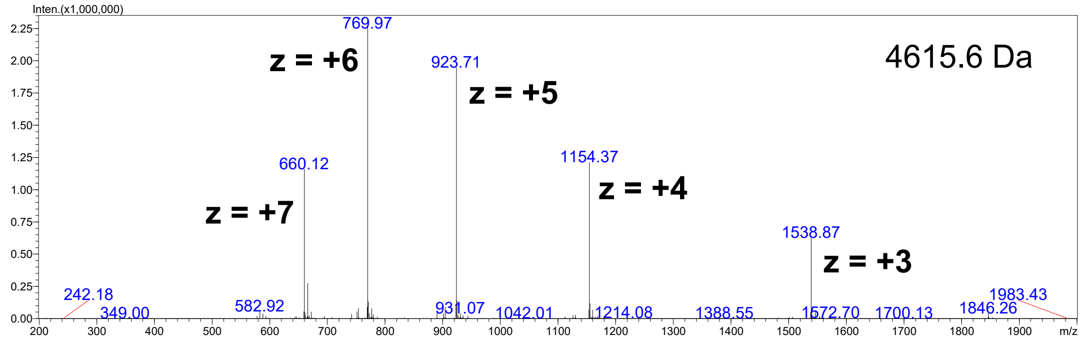

CPT-SS-cPDIP (15) synthesis

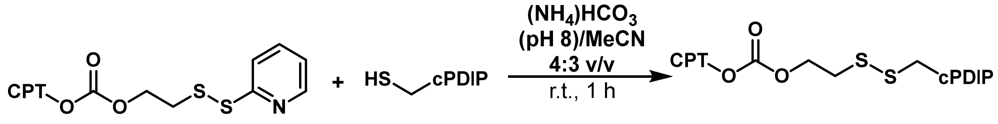

Following a procedure adapted from Zhou *et al.*:[14] cPDIP **2** (2.9 mg, 0.60 µmol, 1.0 equiv.) was dissolved in argon purged H_2O (1 mL). This solution was transferred to a flask containing **5** (0.8 mg, 1.2 µmol, 2.0 equiv.) in argon purged MeCN (1.5 mL), followed by the dropwise addition of an argon purged aqueous ammonium bicarbonate solution (200 mM, pH 8, 1 mL). The mixture was magnetically stirred at room temperature for 1 h, then purified by reverse-phase semi-preparative HPLC (25 to 50% MeCN over 40 min) to afford **15** (1.22 mg, 38%) as a light-yellow solid. Reported yield was determined by weight on a microbalance and was calculated using the mass of the TFA salt of the product (assuming all basic residues and the quinoline moiety within CPT are protonated).

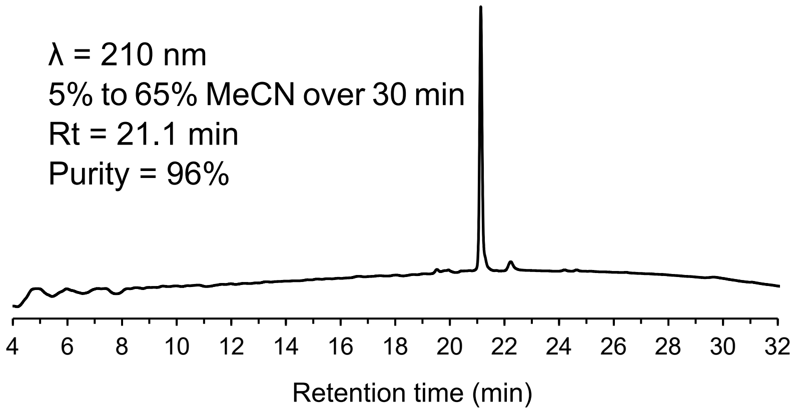

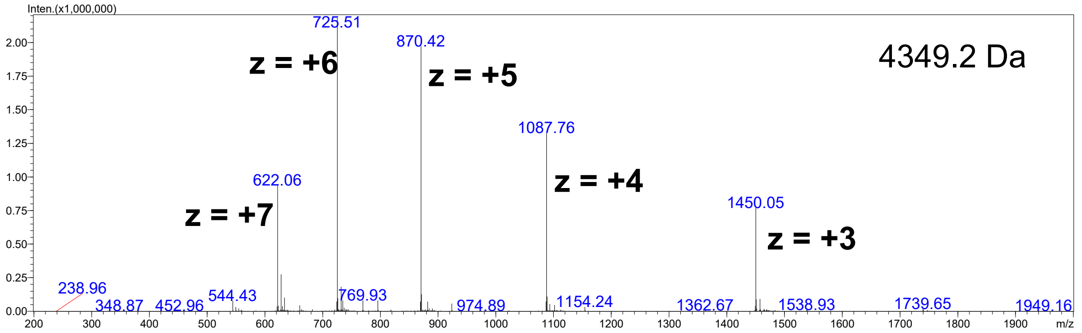

Cell culture

HaCaT cells were grown in DMEM medium and HT144 cells were grown in RPMI medium supplemented with 2 mM L-glutamine and 10 mM sodium pyruvate. Media were supplemented with 10% (v/v) fetal bovine serum, 100 units/mL of penicillin and 100 mg/mL of streptomycin. Flasks of cultured cells were maintained in a humidified incubator (37 °C, 5% CO_2) and passaged every 2–3 days to maintain cultures between 20–90% confluence. Cell line identity was verified by comparing

STR profiles of the cell lines against database entries from the DSMZ-German collection of microorganisms and cell cultures, GmBH https://celldive.dsmz.de/str/browse.

**Marker**	**STR Alleles**	
	**HT144**	**HaCaT**
D5S818	11,13	12
D13S317	11,12	10,12
D7S820	11	9,11
D16S539	12,13	9,12
vWA	16,18	16,17
TH01	9	9.3
Amel	X,Y	X
TPOX	8,11	11,12
CSF1PO	12	9,11

Cell toxicity

Toxicity to the HT144 and HaCaT cell lines was tested following peptide treatment by measuring the metabolism of resazurin into fluorescent resorufin. The assays were performed with 5000 cells/well (96-well plate) in serum free media. Cells were incubated with serially diluted peptides, PDCs or drugs for 72 h or 24 h (37 °C, 5% CO_2). PBS and 0.1% (v/v) Triton X-100 were included as controls for 0% and 100% toxicity. Resazurin was added at 0.02% (w/v) final concentration 2 h prior to the assay end point. Fluorescence (λ_{ex} = 560 nm, λ_{em} = 585 nm) was measured using a Tecan infinite M1000Pro multiplate reader. Percentage cytotoxicity was calculated relative to 0% and 100% controls. Dose-response curves were fitted and CC_{50} values were determined using [inhibitor] *vs* response with four parameters with the top constrained to 100% (GraphPad Prism version 10.0.2). Data was collected as three biological replicates, with experiments conducted on different days.

Red blood cell (RBC) lysis

Human RBCs and serum were obtained from healthy volunteers via the Red Cross Lifeblood, Brisbane, Australia. RBCs were washed three times with PBS and stored in culture medium (RPMI supplemented with 2 mM L-glutamine, 10 mM sodium pyruvate, 2 g/L D-glucose, 52 nM gentamicin, 2.5 g/L Albumax II and 5% human serum). For the lysis assay, RBCs were plated in 96-well plates at 0.25% (v/v) hematocrit in culture medium. Serially diluted compounds were added to the wells, alongside controls including PBS (0% lysis) and 0.1% (v/v) Triton X-100 (100% lysis). The membrane disruptive peptide melittin[15] was also included, starting at 8 µM. The plates were incubated for 72 h (37 ºC, 5% CO_2), then were centrifuged (800 x g, 5 min), before the supernatant was transferred to a new plate. RBC lysis was determined by measuring released hemoglobin at 405 nm using a Tecan infinite M1000Pro multiplate reader. Percentage of lysed RBC was calculated relative to 0% and 100% controls. Data was collected from two technical replicates.

Parallel artificial membrane permeability assay (PAMPA)

PAMPA was performed according to the manufacturer instructions for the BD Gentest pre-coated PAMPA plate system (BD Biosciences), as previously described.[16] Briefly, peptides and PDCs (4 µM in PBS) were added to the apical side of PAMPA wells and incubated for 4 h at 37 °C, 5% CO_2 with 95% humidity. Soluble fractions were recovered from the apical and basolateral sides, and compound concentration was quantified using a Shimadzu LCMS-2020 instrument with a Phenomenex 5 µm C18 / 300 Å / 150 x 2 mm LC column. Data was collected from three technical replicates for each compound. The % recovery was determined from (combined from apical and basolateral fractions) relative to the starting concentration:

$$Recovery\ rate: R\ (\%) = \frac{(C_A \times V_A + C_B \times V_B)}{C_0 \times V_A} \times 100$$

where C_A and C_B are the apical and basolateral concentrations (µM), V_A and V_B are the apical and basolateral volumes (mL), and C_0 is the initial concentration (µM).

The apparent permeability coefficient (Papp) was calculated using the following equations[17] and where A is the membrane surface area (cm^2):

$$Papp\ (cm/s) = \frac{-\ln\left(1 - \frac{C_B}{C_e}\right)}{A \times \left(\frac{1}{V_A} + \frac{1}{V_B}\right) \times time(s)} \qquad C_e = \frac{(C_A \times V_A + C_B \times V_B)}{V_A + V_B}$$

Compound internalization/association with HT144 cells

HT144 cells (5000 cells/well, 96-well plate) were incubated in serum free media with each treatment (final concentration 4 µM) for 1 h (37 °C, 5% CO_2). Media was removed from treated cells, which were washed with PBS, then lysed with ice cold 75% (v/v) MeCN in mQH_2O (containing 1.75% (v/v) TFA) before being transferred to tubes containing PBS (10 µL). Untreated cells were used as controls and were processed in the same way before being transferred to tubes containing an aliquot of each treatment (10 µL, final concentration 4 µM). Cells were incubated for 10 mins at 0 °C (ice), centrifuged (21,000 x g, 4 °C, 20 min) and the supernatant was analyzed using a Qstar elite TOF-MS with a 2% gradient containing 0.1% (v/v) formic acid in H_2O against 0.1% (v/v) formic acid in 90% (v/v) MeCN/ H_2O. The $[M+6H]^{6+}$ *m/z* peak for each compound was identified using Sciex Analyst software, and area under the curve (AUC) was determined for the exact mass using Sciex MultiQuant software. The percentage of internalized peptide was determined from: treated sample AUC / (average of (PBS controls + post-added compound) AUC) × 100% for each peptide analogue. Data was collected from three technical replicates for each treatment and control.

CPT detection inside HT144 cells

HT144 cells were plated and treated with 4 μM of CPT-alk-PDIP (**12**), CPT-SS-DBCO-PDIP (**14**), or CPT, then washed and extracted as above. The recovered supernatant was analyzed using a Sciex QTRAP® 6500+ MS coupled to an Exion UPLC system, with modifications to previously reported quantification methods.[18] Quantification experiments were performed using a Phenomenex Kinetex C18 UPLC column (100 × 2.1 mm, 1.7 μm) at constant temperature of 40 °C or 60 °C using a linear acetonitrile gradient at a flow rate of 0.4 mL min^{-1}. The source setting of electrospray voltage was set at 5500 V, temperature at 600 °C. Targeted multiple reaction monitoring (MRM) scans were performed with low resolution in quadrupole 1 (Q1) and unit resolution in quadrupole 3 (Q3), with the following MRM parameters: Q1 (*m/z*) 349.0, Q3 305.2, collision energy 40, and declustering potential 80. Sciex MultiQuant software was used to integrate the AUC for three technical replicates of each compound.

^{1}H and ^{13}C{^{1}H} NMR

400 MHz ^{1}H NMR in $CDCl_3$

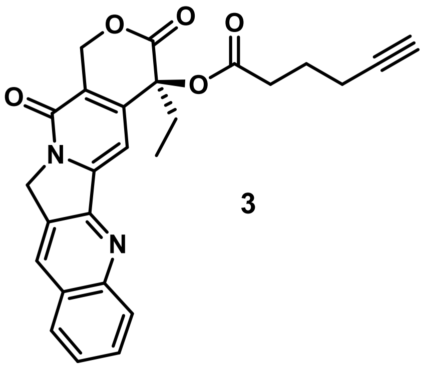

3

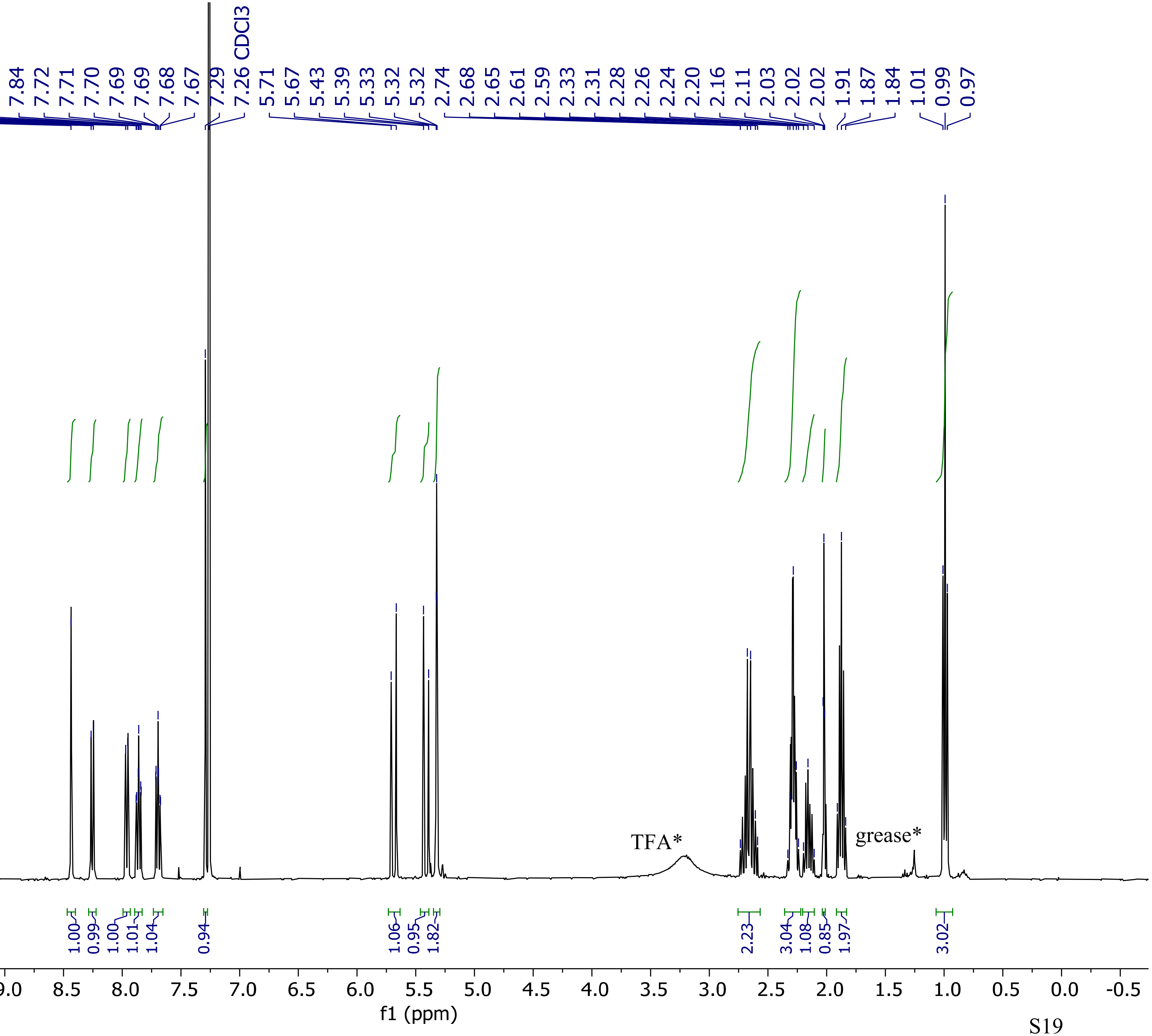

101 MHz ^{13}C NMR in $CDCl_3$

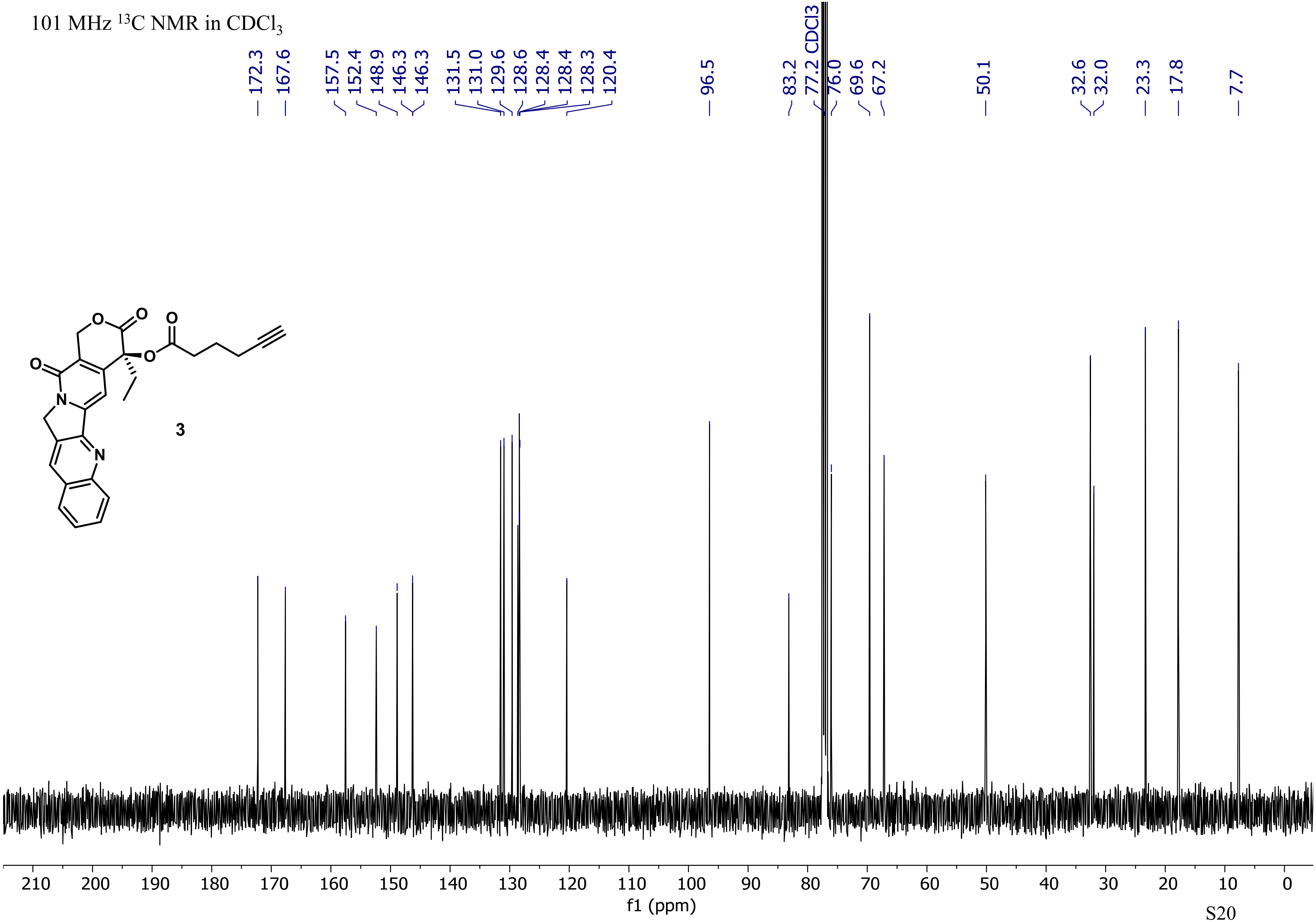

400 MHz ^{1}H NMR in $CDCl_3$

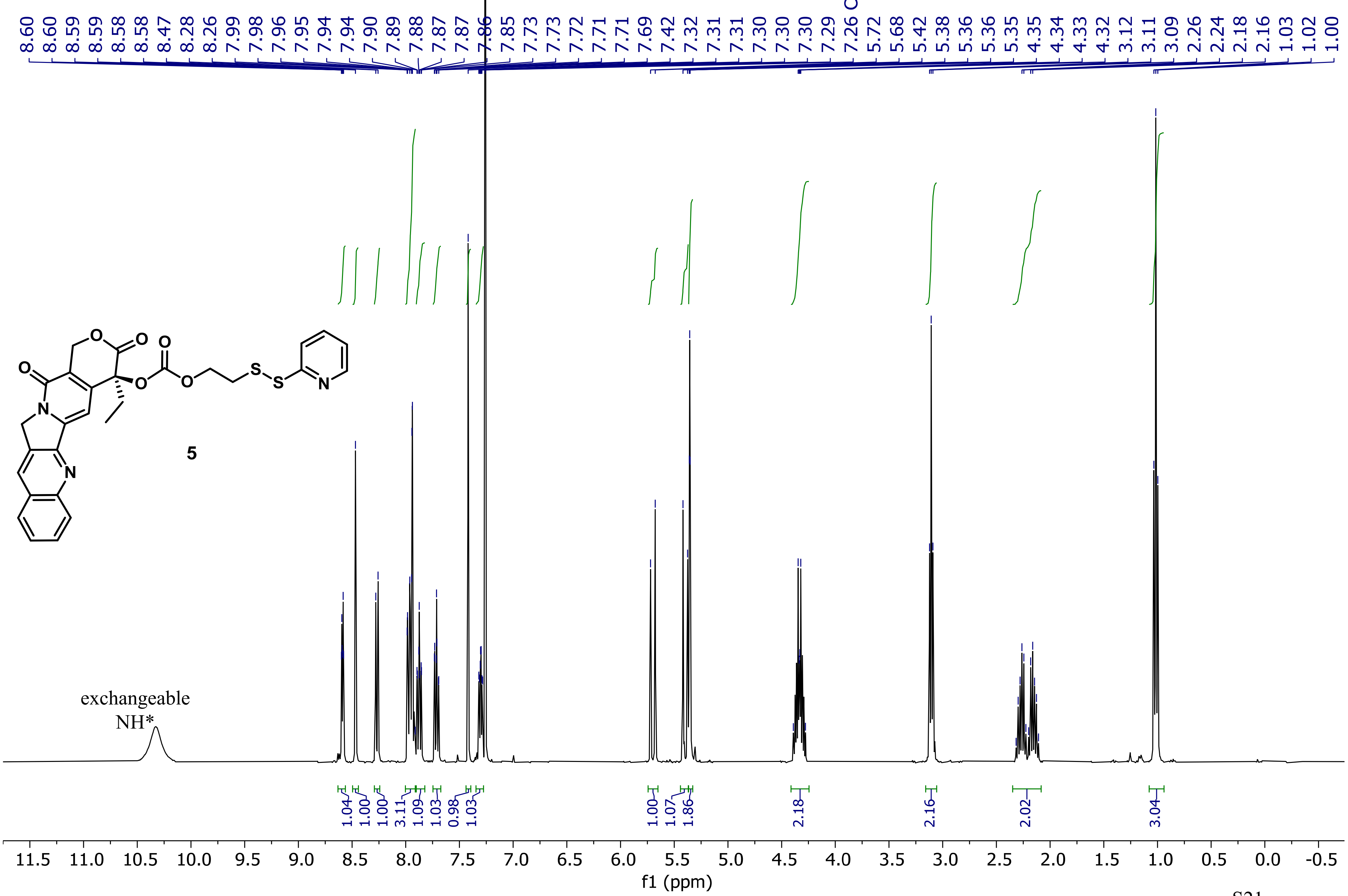

101 MHz ^{13}C NMR in $CDCl_3$

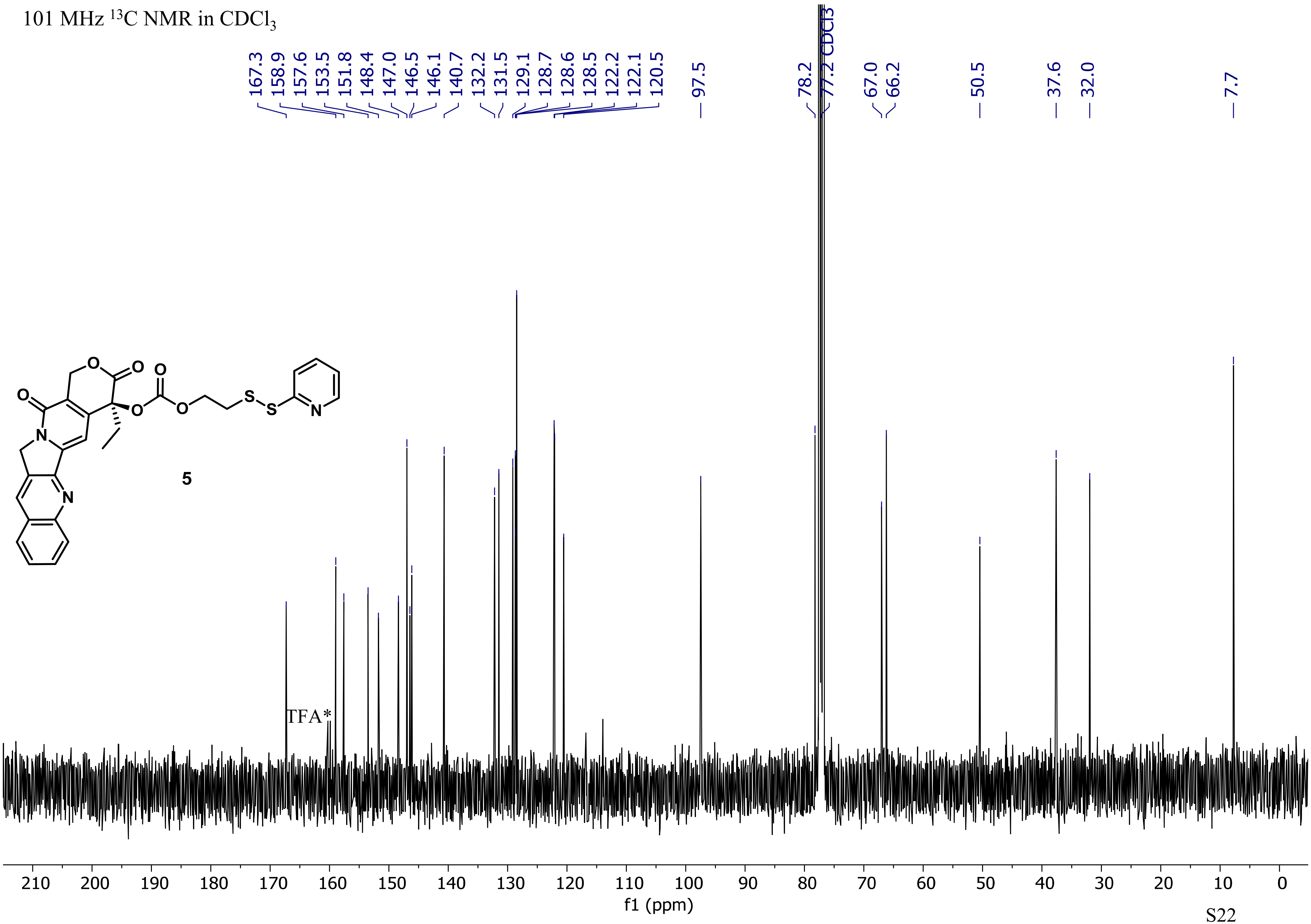

5

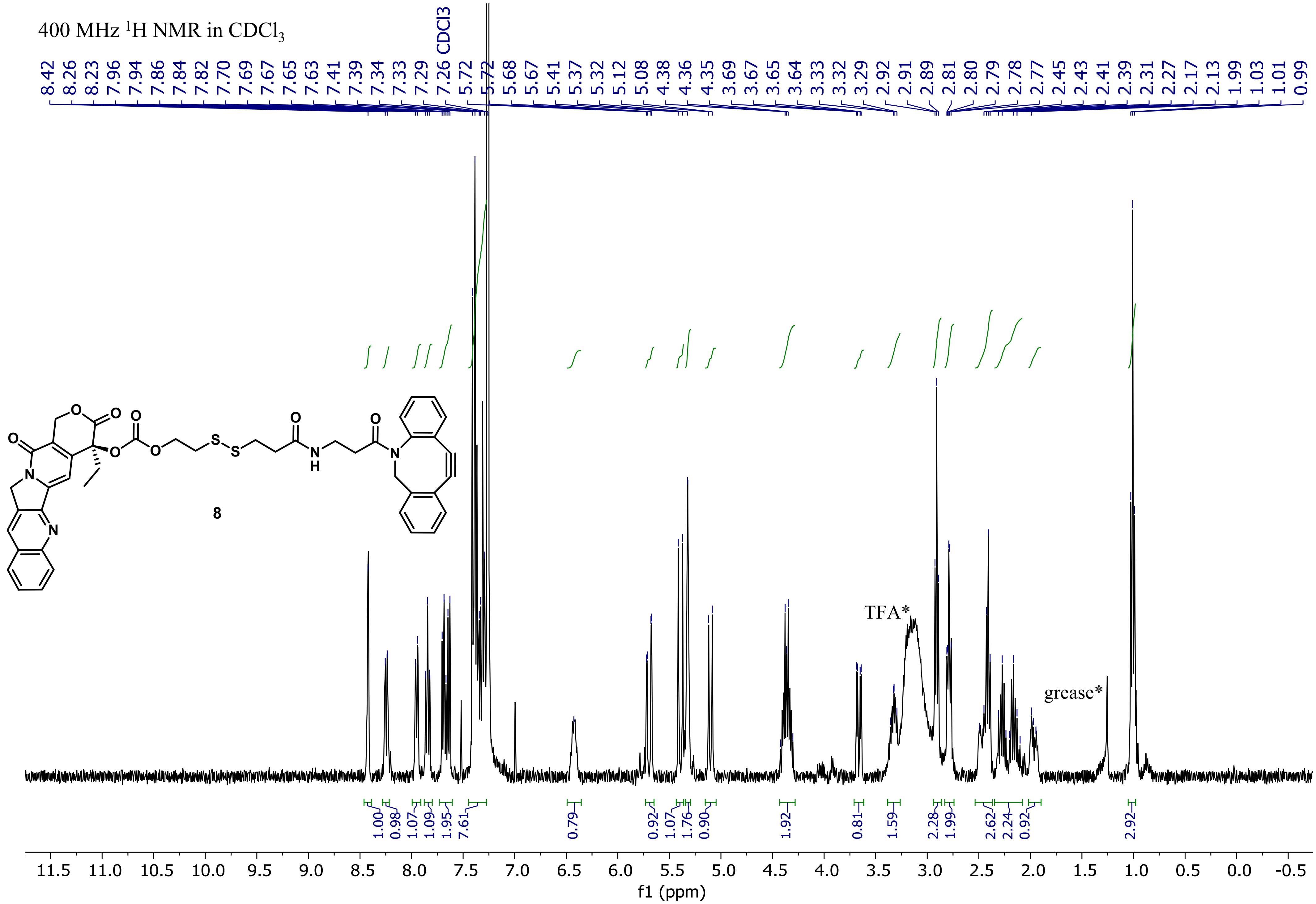
400 MHz ^{1}H NMR in CDCl$_3$
CDCl3
8.42 8.26 8.23 7.96 7.94 7.86 7.84 7.82 7.70 7.69 7.67 7.65 7.63 7.41 7.39 7.34 7.33 7.29 7.26 5.72 5.72 5.68 5.67 5.41 5.37 5.32 5.12 5.08 4.38 4.36 4.35 3.69 3.67 3.65 3.64 3.33 3.32 3.29 2.92 2.91 2.89 2.81 2.80 2.79 2.78 2.77 2.45 2.43 2.41 2.39 2.31 2.27 2.17 2.13 1.99 1.03 1.01 0.99
8
TFA*
grease*
1.00 0.98 1.07 1.09 1.95 7.61 0.79 0.92 1.07 1.76 0.90 1.92 0.81 1.59 2.28 1.99 2.62 2.24 0.92 2.92
11.5 11.0 10.5 10.0 9.5 9.0 8.5 8.0 7.5 7.0 6.5 6.0 5.5 5.0 4.5 4.0 3.5 3.0 2.5 2.0 1.5 1.0 0.5 0.0 -0.5
f1 (ppm)

800 MHz ^{1}H NMR in $CDCl_3$

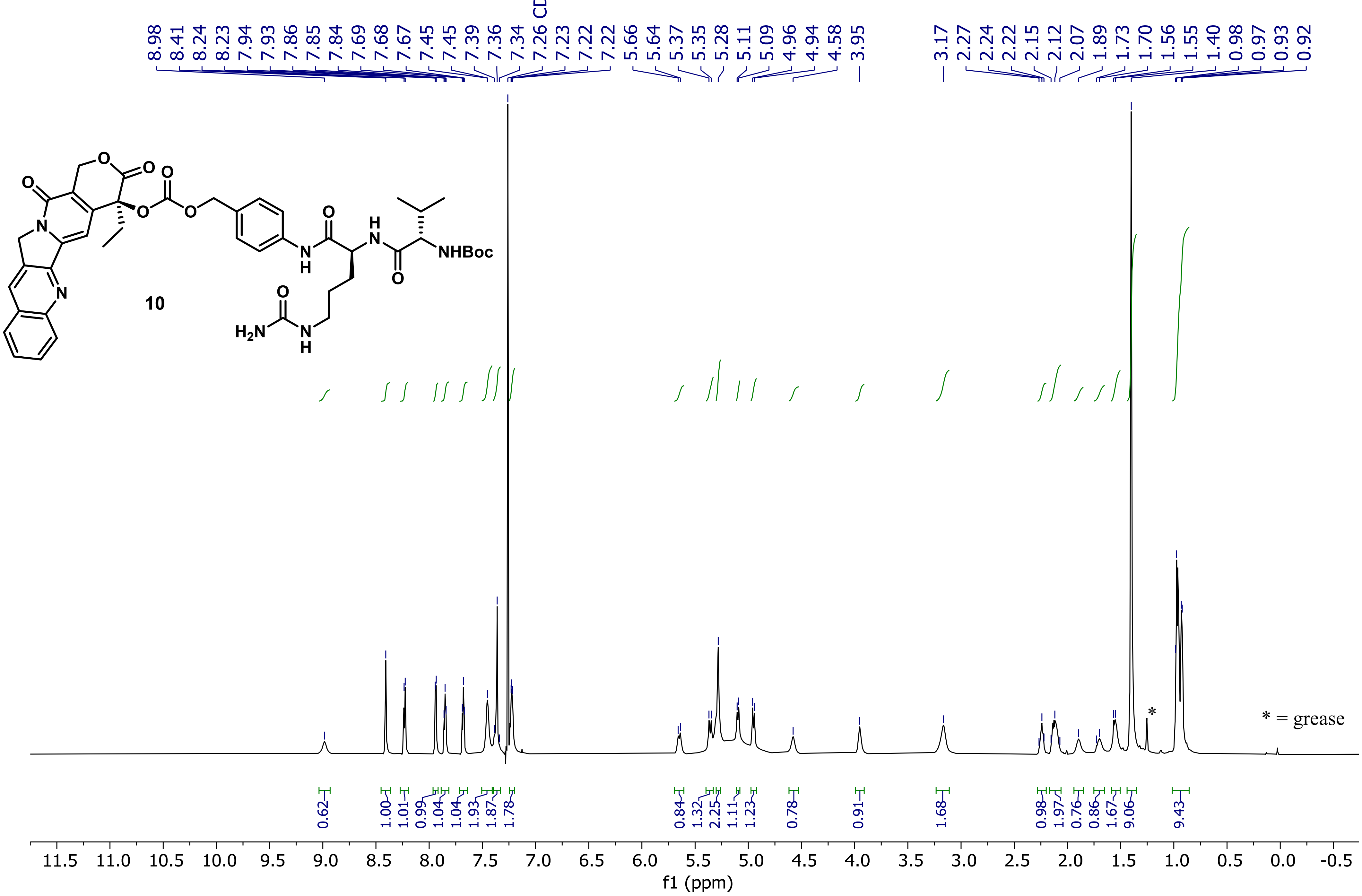

201 MHz ^{13}C NMR in $CDCl_3$

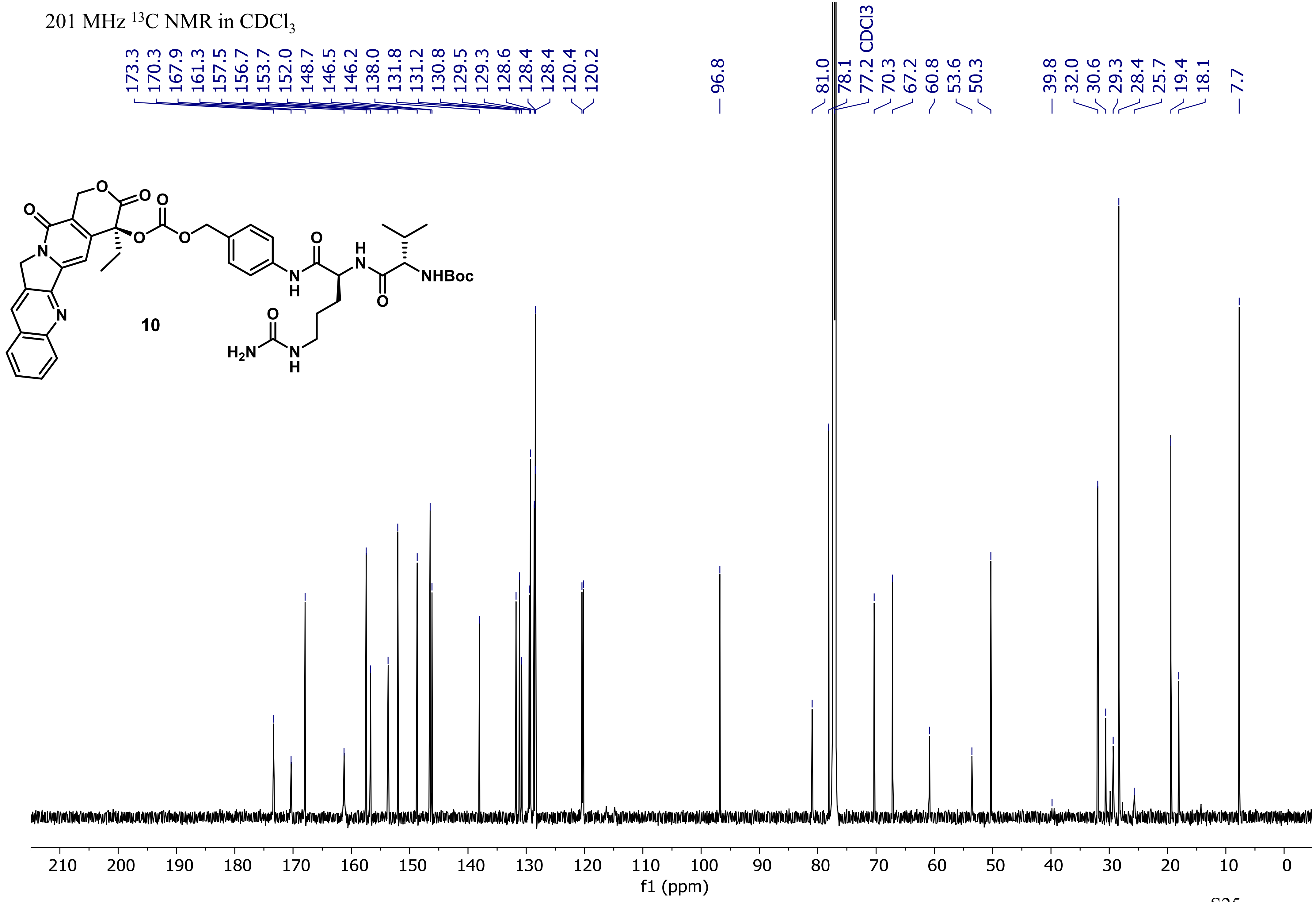

800 MHz, 201 MHz ^{1}H–^{13}C HSQC in $CDCl_3$

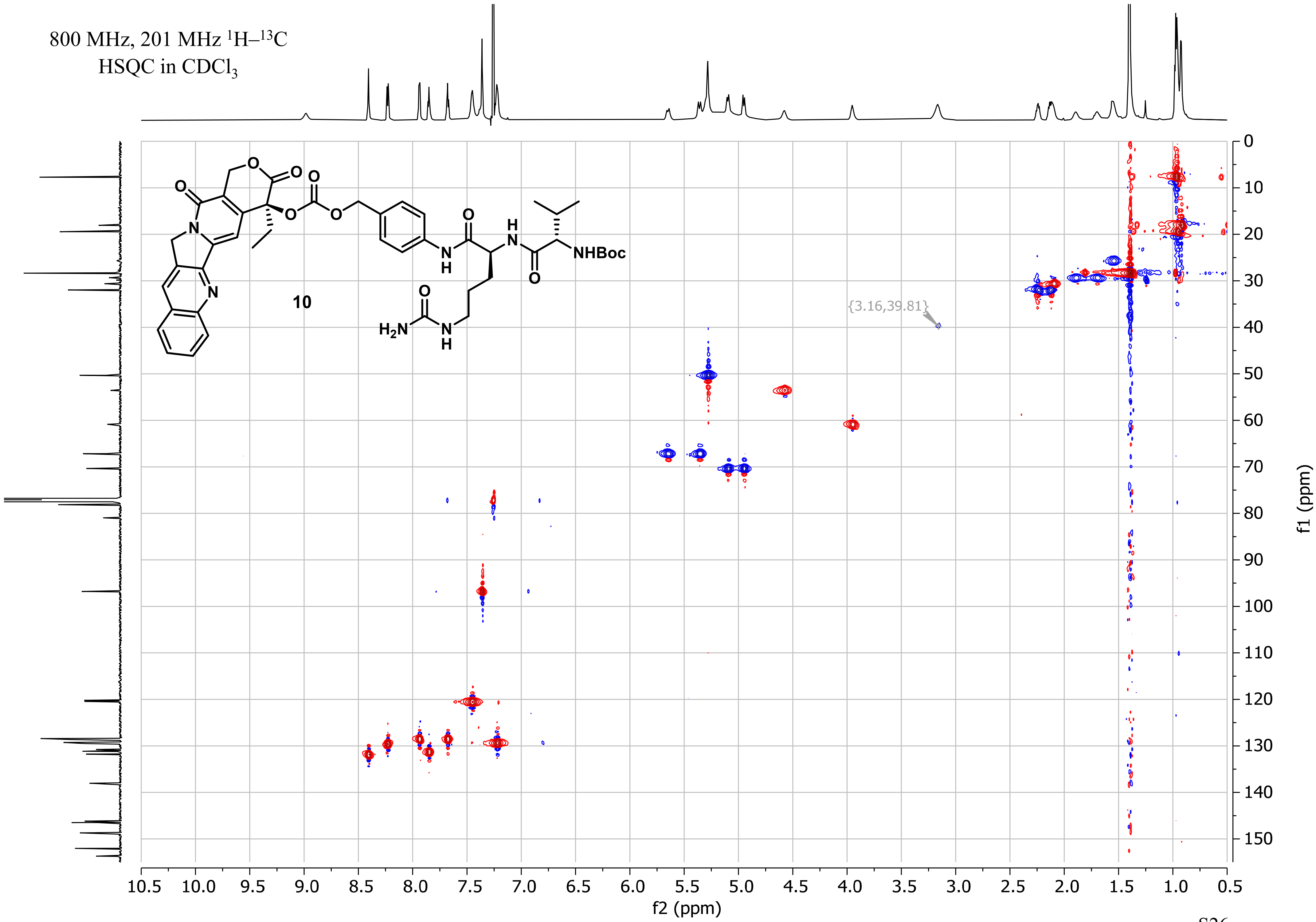

800 MHz ^{1}H NMR in CD_3OD

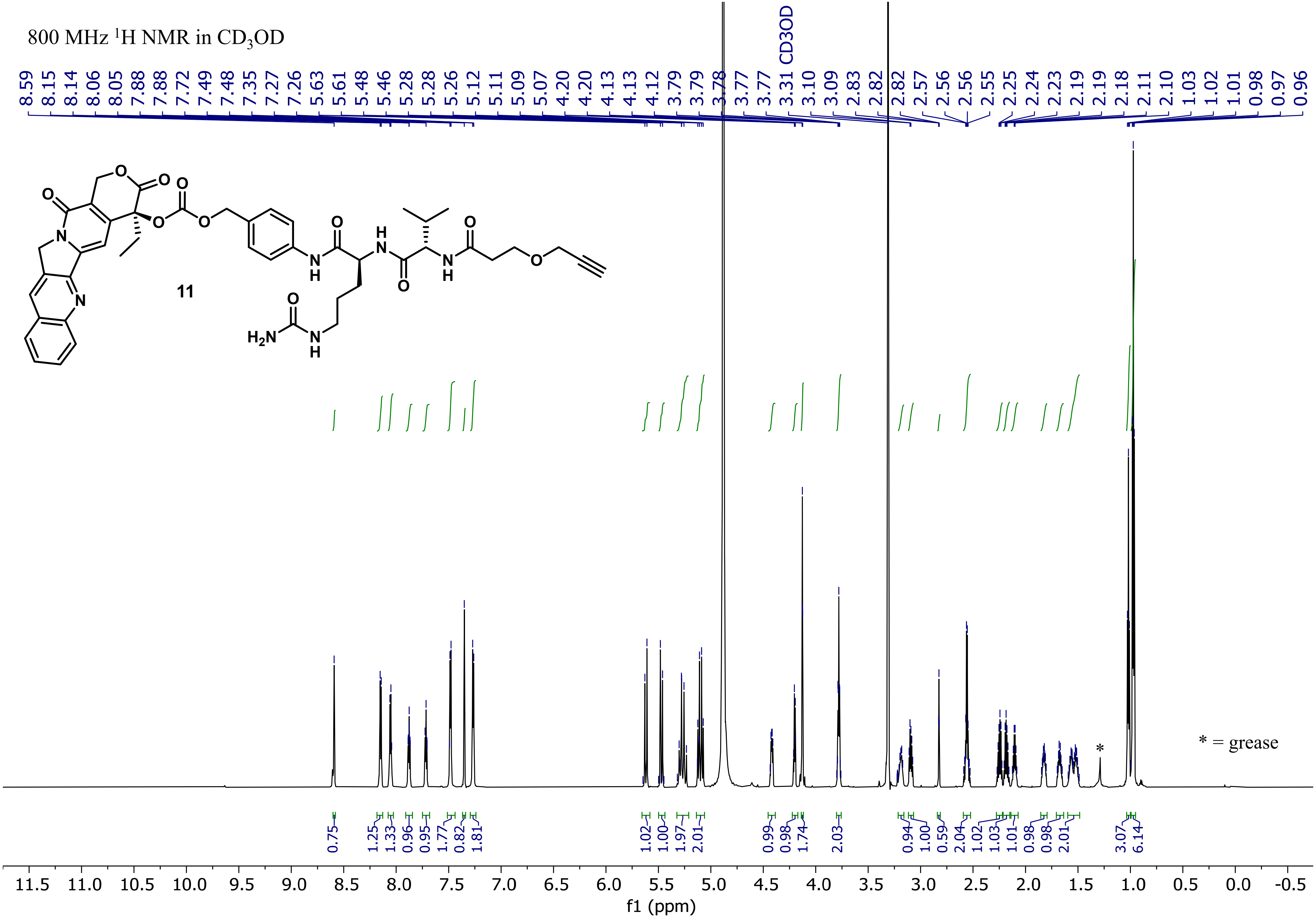

201 MHz ^{13}C NMR in CD_3OD

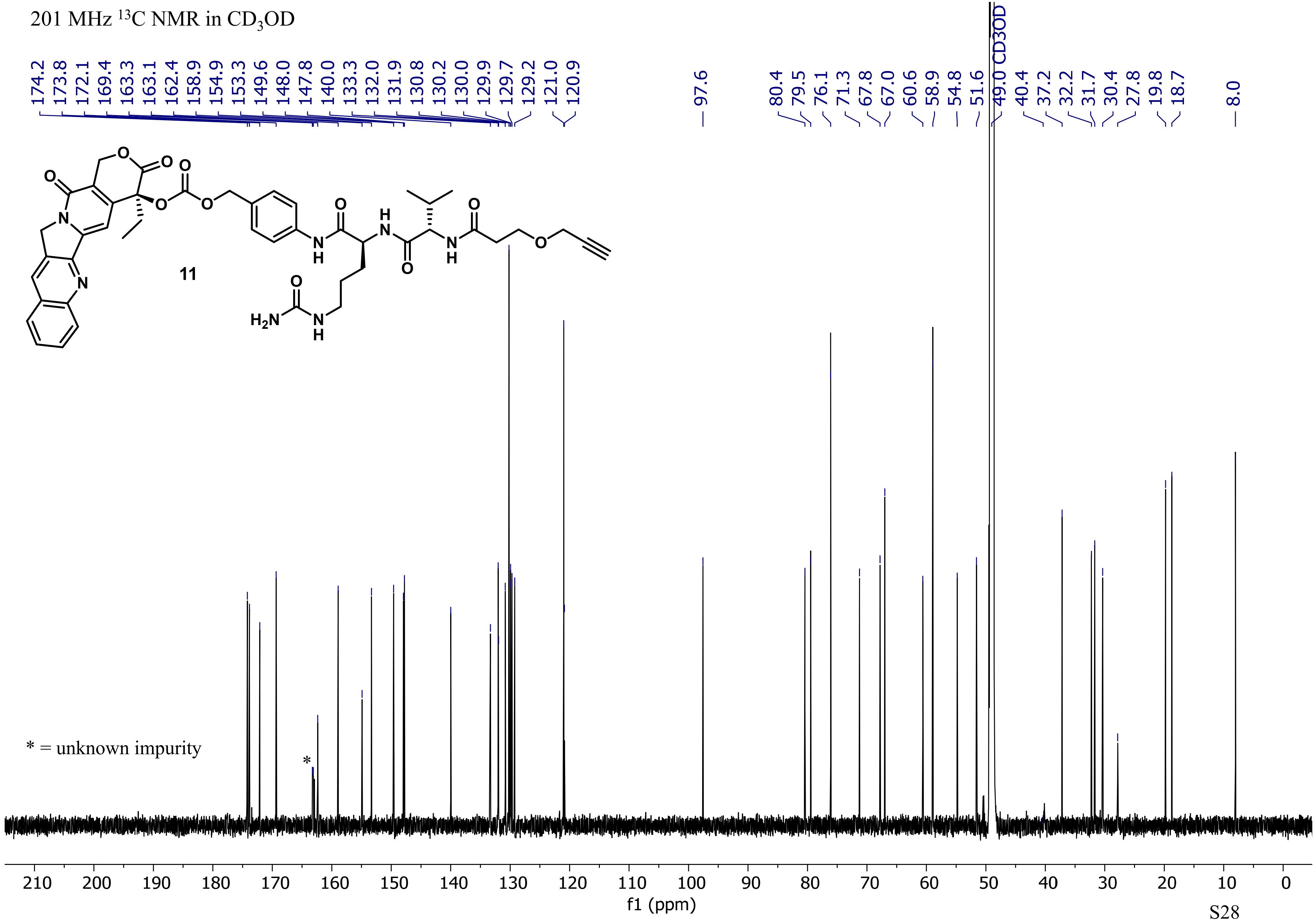

800 MHz, 201 MHz ^{1}H–^{13}C HSQC in $CDCl_3$

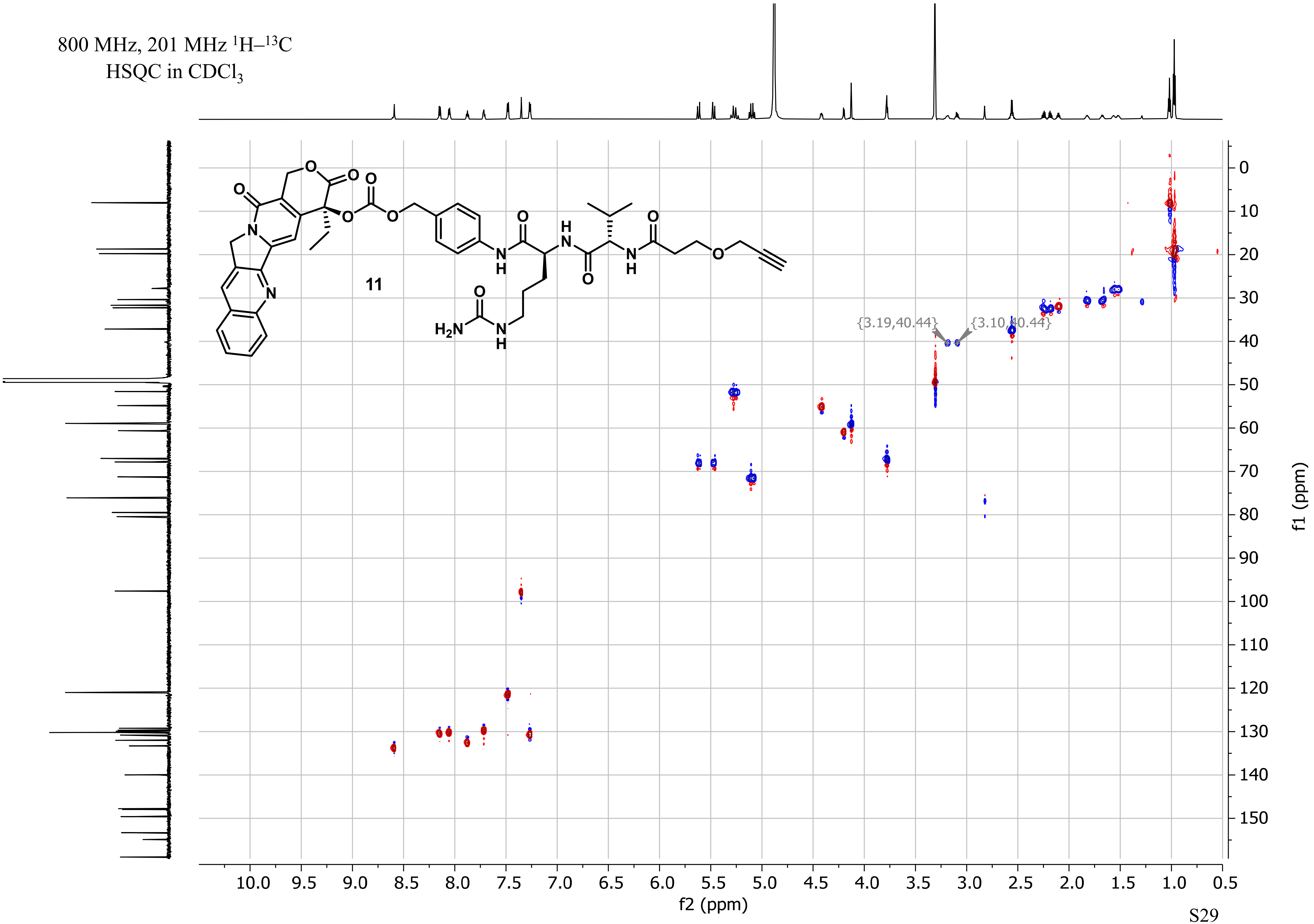

References

(1) Lawrence, N.; Dennis, A. S.; Lehane, A. M.; Ehmann, A.; Harvey, P. J.; Benfield, A. H.; Cheneval, O.; Henriques, S. T.; Craik, D. J.; McMorran, B. J. *Cell Chem. Biol.* **2018**, *25*, 1140–1150.

(2) Lawrence, N.; Philippe, G.; Harvey, P. J.; Condon, N. D.; Benfield, A. H.; Cheneval, O.; Craik, D. J.; Henriques, S. T. *RSC Chem. Biol.* **2020**, *1*, 405–420.

(3) Lawrence, N.; Handley, T. N. G.; de Veer, S. J.; Harding, M. D.; Andraszek, A.; Hall, L.; Raven, K. D.; Duffy, S.; Avery, V. M.; Craik, D. J.; Malins, L. R.; McMorran, B. J. *ACS Infect. Dis.* **2024**, *10*, 2899–2912.

(4) Li, Q.; Lv, H.; Zu, Y.; Qu, Z.; Yao, L.; Su, L.; Liu, C.; Wang, L. *Bioorg. Med. Chem. Lett.* **2009**, *19*, 513–515.

(5) Kularatne, S. A.; Venkatesh, C.; Santhapuram, H. K. R.; Wang, K.; Vaitilingam, B.; Henne, W. A.; Low, P. S. *J. Med. Chem.* **2010**, *53*, 7767–7777.

(6) Palombi, I. R.; Lawrence, N.; White, A. M.; Gare, C. L.; Craik, D. J.; McMorran, B. J.; Malins, L. R. *Bioconjug. Chem.* **2023**, *34*, 1105–1113.

(7) Huang, Y. C.; Chen, C. C.; Li, S. J.; Gao, S.; Shi, J.; Li, Y. M. *Tetrahedron* **2014**, *70*, 2951–2955.

(8) Bird, M. J.; Dawson, P. E. *Pept. Sci.* **2022**, *114*, e24268.

(9) Cistrone, P. A.; Bird, M. J.; Flood, D. T.; Silvestri, A. P.; Hintzen, J. C. J.; Thompson, D. A.; Dawson, P. E. *Curr. Protoc. Chem. Biol.* **2019**, *11*, e61.

(10) Flood, D. T.; Hintzen, J. C. J.; Bird, M. J.; Cistrone, P. A.; Chen, J. S.; Dawson, P. E. *Angew. Chem. Int. Ed.* **2018**, *57*, 11634–11639.

(11) Cergol, K. M.; Thompson, R. E.; Malins, L. R.; Turner, P.; Payne, R. J. *Org. Lett.* **2014**, *16*, 290–293.

(12) Hong, V.; Presolski, S. I.; Ma, C.; Finn, M. G. *Angew. Chem. Int. Ed.* **2009**, *48*, 9879–9883.

(13) Lau, Y. H.; Wu, Y.; Rossmann, M.; Tan, B. X.; De Andrade, P.; Tan, Y. S.; Verma, C.; McKenzie, G. J.; Venkitaraman, A. R.; Hyvönen, M.; Spring, D. R. *Angew. Chem. Int. Ed.* **2015**, *54*, 15410–15413.

(14) Zhou, Y.; Mowlazadeh Haghighi, S.; Liu, Z.; Wang, L.; Hruby, V. J.; Cai, M. *ACS Pharmacol. Transl. Sci.* **2020**, *3*, 921–930.

(15) Asthana, N.; Yadav, S. P.; Ghosh, J. K. *J. Biol. Chem.* **2004**, *279*, 55042–55050.

(16) Bergeron, C.; Bérubé, C.; Lamb, H.; Koda, Y.; Craik, D. J.; Henriques, S. T.; Voyer, N.; Lawrence, N. *Pept. Sci.* **2024**, 10.1002/PEP2.24380.

(17) Sevin, E.; Dehouck, L.; Fabulas-da Costa, A.; Cecchelli, R.; Dehouck, M. P.; Lundquist, S.; Culot, M. *J. Pharmacol. Toxicol. Methods* **2013**, *68*, 334–339.

(18) Yap, K.; Du, J.; Looi, F. Y.; Tang, S. R.; de Veer, S. J.; Bony, A. R.; Rehm, F. B. H.; Xie, J.; Chan, L. Y.; Wang, C. K.; Adams, D. J.; Lua, L. H. L.; Durek, T.; Craik, D. J. *Green Chem.* **2020**, *22*, 5002–5016.
